# Supplementary material for: Genotype-specific differences in infertile men due to loss-of-function variants in M1AP or ZZS genes
Source: EMBO Mol Med. 2025 May 15;17(6):1417–51. doi: 10.1038/s44321-025-00244-0 (PMC12162868; doi:10.1038/s44321-025-00244-0)
Supplement: Supplementary file 1 — Table EV1 [file 44321_2025_244_MOESM1_ESM.docx]

**Table EV1. Clinical parameters of infertile men analysed in this study.**

| **Case** | **Age [y]** | **Origin** | **FSH [U/L]** | **LH [U/L]** | **Testosterone [nmol/L]** | **Testicular volume [mL] – right/left** | **Reference** |
| --- | --- | --- | --- | --- | --- | --- | --- |
| **M1AP** | | | | | | | |
| M330 | 38 | Germany | 9 | 5.3 | 14.6 | 17/23 | Wyrwoll et al., 2020 |
| M864 | 41 | Germany | 4.7 | 1.5 | 9.6 | 19/26 | Wyrwoll et al., 2020 |
| M1792 | 36 | Germany | 7.8 | 5.1 | 10.1 | 15/15 | Nagirnaja et al., 2022; Wyrwoll et al., 2020 |
| M2062 | 26 | Poland | 3.5 | 3.6 | 18.6 | 26/23 | Wyrwoll et al., 2023, 2020 |
| M2525 | 43 | Germany | 15.4 | 7.7 | 10.2 | 22/29 |  |
| M2746 | 29 | Germany | 3.5 | 2.4 | 18.5 | 18/16 |  |
| M2747 | 33 | Germany | 9.7 | 3.6 | 19.9 | 8/8 |  |
| M3402 | 43 | NA | 7.2 | 5.2 | 10.7 | 15/14 |  |
| M3511 | 41 | Germany | 3 | 2.1 | 8.9 | 22/13 |  |
| M3609 | 35 | Germany | 3.8 | 3.7 | 17.1 | 10/10 |  |
| **SHOC1** | | | | | | | |
| M2012 | 23 | Iraq | 5.9 | 4.6 | 17.1 | 23/19 | Krausz et al., 2020 |
| G-377 | 27 | Denmark | 4.9 | 7.4 | 29.6 | NA | Nagirnaja et al., 2022 |
| M2046 | NA | NA | 15.9 | 10.1 | 6.3 | 10/10 | Krausz et al., 2020 |
| M3260 | 32 | India | 4.8 | 5.2 | 11.9 | 18/13 |  |
| **TEX11** | | | | | | | |
| M205 | 52 | Germany | 6 | 2.5 | 15.1 | 25/5* | Yatsenko et al., 2015 |
| M246 | 41 | Germany | 3.3 | 4.1 | 10.9 | 26/22 |  |
| M281 | 54 | NA | 2.9 | 2.2 | 12.8 | 20/16 | Yatsenko et al., 2015 |
| M1390 | 42 | Germany | 11.6 | 5.9 | 13.1 | 12/10 | Wyrwoll et al., 2023 |
| M2739 | 35 | Germany | 7.3 | 3.1 | 15.8 | 28/28 |  |
| M2820 | 33 | Germany | 15.9 | 5.2 | 10.4 | 14/5^+^ |  |
| M2942 | 33 | Afghanistan | 5.5 | 2 | 14 | 15/17 |  |
| M3152 | 32 | Turkey | 5.3 | 3.6 | 22 | 11/10 |  |
| M3409 | 35 | NA | 7.1 | 5.3 | 8.9 | 10/10 |  |
| **SPO16** | | | | | | | |
| M3609 | NA | NA | 5.7 | 4.1 | 14.2 | 11/13 |  |
| abbreviations: FSH = follicle-stimulating hormone, LH = luteinising hormone, NA = not available. All parameters were obtained at the first visit. *diagnosed and treated varicocele at the age of 18, led to testis atrophy (left side), ^+^maldescended testis (left side).  reference values: FSH = 1-7 IU/L, LH = 2-10 IU/L, T = >12 nmol/L, TV = >12 mL per testis (right/left). | | | | | | | |
