# Supplementary material for: Genotype-specific differences in infertile men due to loss-of-function variants in M1AP or ZZS genes
Source: EMBO Mol Med. 2025 May 15;17(6):1417–51. doi: 10.1038/s44321-025-00244-0 (PMC12162868; doi:10.1038/s44321-025-00244-0)
Supplement: Supplementary file 2 — Appendix [file 44321_2025_244_MOESM2_ESM.pdf]

Appendix

Genotype-specific differences in infertile men due to loss-of-function variants in *M1AP* or *ZZS* genes

Nadja Rotte[1], Jessica E.M. Dunleavy[2], Michelle D. Runkel[1], Lina Bosse[1], Daniela Fietz[3], Adrian Pilatz[4], Johanna Kuss[1], Ann-Kristin Dicke[1], Sofia B. Winge[7], Sara Di Persio[5], Christian Ruckert[6], Verena Nordhoff[5], Hans-Christian Schuppe[4], Kristian Almstrup[7,8], Sabine Kliesch[5], Nina Neuhaus[5], Birgit Stallmeyer[1], Moira K. O'Bryan[2], Frank Tüttelmann[1], Corinna Friedrich[1]

Table of Content

Appendix Tables....4
Appendix Table S1. Published cases of male infertility due to LoF variants in M1AP, SHOC1 or TEX11. 4
Appendix Table S2. Primer information....8
Appendix Table S3. Antibody information. 11
Appendix Table S4. ClinVar accession numbers of the variants in *M1AP, SHOC1, TEX11,* and *SPO16*....13
Appendix Figures 14
Appendix Figure S1. Digital droplet PCR confirmed the deletion of TEX11 exons 1-11 in M2820. 14
Appendix Figure S2. Digital droplet PCR confirms the deletion of TEX11 exons 10-11 in M3152. 15
Appendix Figure S3. Overview of clinical data of men with loss-of-function variants in M1AP, SHOC1, TEX11 or SPO16. 16
Appendix Figure S4. PAS (N=7) or H&E (N=3) staining of men with LoF variants in M1AP..17
Appendix Figure S5. PAS (N=6) or H&E (N=3) staining of men with LoF variants in SHOC1, TEX11 or SPO16....18
Appendix Figure S6. CREM staining in men with loss-of-function variants in M1AP, SHOC1, TEX11 or SPO16....19
Appendix Figure S7. γH2AX localisation showed meiosis prophase I progression in men with loss-of-function variants in M1AP. 21
Appendix Figure S8. γH2AX localisation showed impaired meiosis prophase I progression in men with loss-of-function variants in SHOC1, TEX11 or SPO16. 22

Appendix Figure S9. Metaphase I cells were determined by H3S10p localisation in men with loss-of-function variants in M1AP, SHOC1, TEX11 or SPO16...23

Appendix Figure S10. Immunohistological staining of H3S10p, yH2AX, and MAGEA4 of sequential testicular sections with a distance of 3 µm...25

Appendix Figure S11. Germ cell apoptosis in men with loss-of-function variants in M1AP, SHOC1, TEX11 or SPO16. 26

Appendix Figure S12. Staining of testicular tissue from a representative human control. 27

Appendix Reference..28

43 **Appendix Tables**

44 **Appendix Table S1. Published cases of male infertility due to LoF variants in M1AP, SHOC1 or TEX11.**

Case #	Case ID	LoF variant		Genotype	Phenotype	Transcript	Reference
**M1AP**							
1	M330	c.676dup	p.Trp226Leufs*4	1/1	azoo (MeiA – SPC), TESE negative	NM_138804.4	Wyrwoll et al., 2020
2	M864				azoo (MeiA – SPC), TESE negative	NM_138804.4	Wyrwoll et al., 2020
3	M1792				azoo (MeiA – SPC), TESE negative	NM_138804.4	Nagirnaja et al., 2022; Wyrwoll et al., 2020
4	M2062				crypto (MeiA – ES)	NM_138804.4	Wyrwoll et al., 2023, 2020
5	RU01691				azoo (MeiA – postmeiotic cells), TESE positive	NM_138804.4	Wyrwoll et al., 2020
6	MI-0006-P				azoo (MeiA – RS [note: not validated by CREM IHC]), TESE negative	NM_138804.4	Wyrwoll et al., 2020
7	F1: II-1	c.1435-1G>A	p.?	1/1	severe oligozoospermia	NM_138804	Tu et al., 2020
8		c.1074+2T>C	p.Ala312Lysfs*7	1/1	azoo (M-I arrest – RS [note: not validated by CREM IHC])	NM_001321739.2	Li et al., 2023

9	9-azoo	c.(*142767)_(*153120)del	p.?	1/1	azoo	NM_001281296.2	Khan et al., 2023
10	GEMINI-1678	c.676dup	p.Trp226Leufs*4	1/1	severe oligozoospermia	NM_001281296.2	Khan et al., 2023
11	GEMINI-283	c.676dup	p.Trp226Leufs*4	1/1	NOA	NM_001281296.2	Khan et al., 2023
**SHOC1**							
1	11-272	c.797del	p.(Leu266Glnfs*6)	1/1	SPC arrest, M-I	NM_173521.4	Krausz et al., 2020
2	M2046	c.[1351del;1347T>A];[945_948del]	p.([Ser451Leufs*23;Cys449*];[Glu315Aspfs*6])	1/1	MA	NM_173521.4	Krausz et al., 2020; this study
3	M2012	c.1085_1086del	p.(Glu362Valfs*25)	1/1	azoo (MA)	NM_173521.4	Krausz et al., 2020; Wyrwoll et al., 2023; this study
4	Family 1	c.1582C>T c.231_232del	p.(Arg528*) p.(Leu78Serfs*10)	1/1	MA (SPC)	NM_173521	Yao et al., 2021
5	Family 2	c.1194del	p.(Leu400Cysfs*8)	1/1	MA	NM_173521	Yao et al., 2021
6	sporadic MA	c.1464del	p.(Asp489Thrfs*14)	1/1	MA	NM_173521	Yao et al., 2021
7	F1:II-2	c.231_232del	p.(Leu78Serfs*10)	1/1	MA (SPC)	NM_173521	Wang et al., 2022
8	F2:II-1	c.231_232del	p.(Leu78Serfs*10)	1/1	MA (SPC)	NM_173521	Wang et al., 2022

9	GEMINI-377	c.1085_1086del	p.(Glu362Valfs*25)	1/1	NA	NM_173521.4	Nagirnaja et al., 2022; this study
TEX11							
1	Patient 1	c.(651+1_652-1)_(888+1_889-1)del		1/-	mixed testicular atrophy	NM_001003811	Yatsenko et al., 2015
2	Patient 3				MeiA	NM_001003811	Yatsenko et al., 2015
3	Patient 4	c.1837+1G>C	p.?	1/-	MeiA (RS [note: not validated by CREM IHC])	NM_001003811	Yatsenko et al., 2015
4	Patient 5	c.792+1G>A	p.?	1/-	MeiA	NM_001003811	Yatsenko et al., 2015
5	WHT3759	c.1259_1260insTT	p.(Trp421Cysfs*25)	1/-	MA (pachytene)	NM_031276	Yang et al., 2015
6	WHT2445	c.1793-1G>A	p.?	1/-	azoo	NM_031276	Yang et al., 2015
7	09-297	c.(82+1_83-1)_(651+1_652_1)del		1/-	SPC arrest, M-I	NM_001003811.2	Krausz et al., 2020
8	NOA8	c.2525G>A	p.(Trp842*)	1/-	MA	NM_001003811.1	Chen et al., 2020
9		c.151_154del	p.(Asp51Phefs*8)	1/-	MeiA (RS [note: not validated by CREM IHC])	NM_031276	Yu et al., 2021
10	P5648	c.1796+2T>G	p.?	1/-	MA	NM_001003811	Ji et al., 2021

11	P6825	c.1426-1G>T	p.?	1/-	MA	NM_001003811	Ji et al., 2021
12	P8122	c.1253dup	p.(Asn418Lysfs*10)	1/-	MA	NM_001003811	Ji et al., 2021
13	P8251	c.298del	p.(Val100Leufs*6)	1/-	NOA	NM_001003811	Ji et al., 2021
14	P5048	c.1051G>T	p.(Glu351*)	1/-	MA	NM_001003811	Ji et al., 2021
15	P9225	c.857del	p.(Lys286Argfs*6)	1/-	NOA	NM_001003811	Ji et al., 2021
16	A2799	c.2240C>A	p.(Ser747*)	1/-	MeiA	NM_031276	An et al., 2021
17	A2153	c.1246C>T	p.(Gln416*)	1/-	NOA	NM_031276	An et al., 2021
18	NOA49	c.559_560del	p.(Met187Valfs*6)	1/-	MA	NM_031276	Tang et al., 2022
19	P3	c.313C>T	p.(Arg105*)	1/-	MA, TESE negative	NM_031276	Song et al., 2023
20	M1390	c.(159+1_160-1)_(692+1_693-1)del		1/-	MeiA	NM_001003811.2	Wyrwoll et al., 2023

abbreviations: c = coding DNA reference sequence, p =protein reference sequence, azoo = azoospermia, crypto = cryptozoospermia, MeiA, meiotic arrest, M-I = metaphase I arrest, MA = maturation arrest, RS = round spermatid, SPC = spermatocyte, NA = no information, NOA = non-obstructive azoospermia, TESE = testicular sperm extraction

Appendix Table S2. Primer information.

Target	Primer sequences (5' – 3')
**Sanger sequencing**	
M1AP c.676dup	F: TGGGTCTGGAAATGTTGCTGA<br>R: GATTGCTAGAGCCCAGGCAT
M1AP c.1073_1074+10del	F: ACAGAATATATATCTAGGGCTTGACAC<br>R: GAGTCTGCTTCAACTCTTCCCA
SHOC1 c.1085_1086del	F: GCAGAGCCAGGGCCTATATG<br>R: AATTCAAGAGCCCCACAGCC
SHOC1 c.1351del + c.1347T>A	F: TGGTCCTGTGCAGTCAAGTT<br>R: ACACTCGTTTAGGTGTGGAAGT
SHOC1 c.945_948del	F: CCCACATTTCTACTCGTTGTACC<br>R: CTTGAATCTGGGGCGGAGG
SHOC1 c.1939+2T>C	F: TGTCAATTAGGAGCTTCACTGAG<br>R: TGCAGATAGCCAGTGCCAA
TEX11 c.450C>T	F: TGTGGAGTTCAAAGTAGAACACAGAAC<br>R: TCCAATCAGCATTAGTAACATCACC
TEX11 c.1425G>A	F: TGTTGACCAAGACTGATAATAAAATGC<br>R: CAGTGTGCAAATCAAGAAAATGTC
TEX11 c.22del	F: CGTTGCCAGGCAGACTTATG<br>R: GGGATTACCACGCCCAAC
TEX11 c.1096dup	F: CACTCTCCAGCACTGGATGTTAATAC<br>R: ATTGCCAAGGTTGGTCTCAAG
TEX11 c.792+1G>A	F: GCCAAATGGAAAAAGGCATC<br>R: ACCCAAACATTGTTCAAAGCAC
TEX11 c.1837+1G>C	F: ATGAGGGCACTGGGAATGAG<br>R: TCTCTGCTTGTGAATGAAGAAACC
TEX11 c.1245G>A	F: GGAGAATCAGGCAGCAGTACC<br>R: CAGCGATGACATTTCCCTACAC

TEX11 c.731G>A	F: TTCTCCAACCTGAATGTTTTGC<br>R: AAGGAAGGAAGAACACATTTTTCTATG
SPO16 Exon 4	F: ACTACCCAGTATCTTCATGTGGA<br>R: CCACAGAGGATTTGAGATGGCT
pcDNA3.1	F: GTAACAACTCCGCCCCATTG<br>R: AGGAAAGGACAGTGGGAGTG
SHOC1 WT (cDNA)	F: GCCAAGAATTCAAGAGCCCC<br>R: CCTGCTTGTTTCCACCACTC
SHOC1 WT c.287 (cDNA)	F: GTAGTAGAAAACACCTACC
SHOC1 WT c.803 (cDNA)	F: CTCTATTCCTAACATGCC
SHOC1 WT c.1324 (cDNA)	F: GCAAAAGAAGTACCAGATC
SHOC1 WT c.1979 (cDNA)	F: CTCTCTTACATCTTCTGG
SHOC1 WT c.2636 (cDNA)	F: CAGACATACTTCAGCTGC
SHOC1 WT c.3086 (cDNA)	F: GGTTGGATAAATCCTGGC
SHOC1 WT c.3746(cDNA)	F: CTCAGAAGAGAGTGTCAG
SHOC1 WT c.4241 (cDNA)	F: TGTGCTCACAACTACCAC
TEX11 WT (cDNA)	F: TGGCCTTGCGTTTCCTTAAC<br>R: ACTGGGCCCTTGTTGTTACT
TEX11 WT c.276 (cDNA)	F: AAGCCTCATTTGCCTCAG
TEX11 WT c.809 (cDNA)	F: ATAAGGCTCTCAATGCTG
TEX11 WT c.1404 (cDNA)	F: TGAACGACATGACCCTAG
TEX11 WT c.2021 (cDNA)	F: CAGTTGATCTAGAGCAAG
TEX11 WT c.2700 (cDNA)	F: TAGTCAGCTTGTGGAAGC
SPO16 WT (cDNA)	F: GTTTTGTCTGCTGCCCTCC<br>R: GCATTTACTGTGTTGTGTACTGG
SPO16 WT c.355 (cDNA)	F: CTTCCAGTACACAACACAG
**ddPCR**	
TEX11 WT	F: TGGGTTAACTGTGAGGAGAC<br>R: CAATTCTCCCCTCTCCCTAC

TEX11 (probe)	FAM-ACGCTGAGTGAAACAAGCCAGTC-BHQ1
Reference WT (ZIC1)	F: CTCTGGCTACGAATCCTCC<br>R: CAATTCTCCCCTCTCCCTAC
Reference (ZIC1, probe)	HEX- CGCCTCCCACCATCGTGTCT
**Minigene assay**	
M1AP in Ex7 F-seq	F: CACCTCTGCTTCAACTCTTCCCAGT<br>R: TACACCTGGAATGCTCTGCC
M1AP aus Ex7 R-seq	F: TGTAAAACGACGGCCAG<br>R: AGCAGGCTGTGACACAAAGCATG
SHOC1 c1339+2 MiniGene	F: CACCTTCAGATAGAAGTTCGGATCTCC<br>R: TGGTTTGGCTGGGTATCACA
SHOC1 Ex13	F: ACCCTCCCTACTGCTAATTGG
rat insulin	F: CCTGCTCATCCTCTGGGAGC<br>R: AGCAGGCTGTGACACAAAGCATG

48 **Appendix Table S3. Antibody information.**

Antibodies	Reference or Source	Identifier or Catalog Number	Dilution, individual protocol requirements
**primary**			
goat anti-SYCP3	R&D Systems, Minneapolis, USA	AF3750	IF (meiotic spreads): 1:500
human anti-ACA	Biozol, Eching, Germany	15-234	IF (meiotic spreads): 1:150
mouse anti-DYK	Merck, Darmstadt, Germany	F3165	WB: 1:1500
mouse anti-γH2AX	Merck, Darmstadt, Germany	05-636	IHC: 1:30 in TBS + 0.01% Tween, pH 9<br>IF (meiotic spreads): 1:1000
mouse anti-MAGEA4	abcam, Cambridge, United Kingdom	ab139297	IHC: 1:500
mouse anti-MLH1	BD Bioscience, Franklin Lakes, USA	550838	IF (meiotic spreads): 1:25
mouse anti-RAD51	Santa Cruz, Dallas, USA	sc-398587	IF (meiotic spreads): 1:300
rabbit anti-CREM	Sigma Aldrich, St. Louis, USA	HPA001818	IHC: 1:2000 pH 6
rabbit anti-HA	Sigma Aldrich, St. Louis, USA	11867423	WB: 1:1500
rabbit anti-H3S10p	GeneTex, Irvine, USA	GTX128116	IHC: 1:2500, pH 9
rabbit anti-MSH5	Sigma Aldrich, St. Louis, USA	HPA062688	IF (meiotic spreads): 1:20
rabbit anti-SYCP1	Novus Biological, Minneapolis, USA	NB300-228	IF (meiotic spreads): 1:50
rabbit anti-TEX11	Sigma Aldrich, St. Louis, USA	HPA002950	IF (meiotic spreads): 1:25, WB: 1:500
**secondary**			
donkey anti-goat Alexa Fluor 488	Thermo Scientific, Waltham, USA	A11055	IF (meiotic spreads): 1:500
donkey anti-human DyLight 405	Jackson ImmunoResearch, West Grove, USA	709-475-149	IF (meiotic spreads): 1:400
donkey anti-mouse Alexa Fluor 568	Thermo Scientific, Waltham, USA	A10037	IF(meiotic spreads): 1:500
donkey anti-mouse Alexa Fluor Plus 647	Thermo Scientific, Waltham, USA	A32787	IF(meiotic spreads): 1:500
donkey anti-mouse HRP	Santa Cruz, Dallas, USA	sc-516102	WB: 1:1000
donkey anti-rabbit Alexa Fluor 568	Thermo Scientific, Waltham, USA	A10042	IF (meiotic spreads): 1:500
donkey anti-rabbit Alexa Fluor Plus 647	Thermo Scientific, Waltham, USA	A32795	IHC: 1:100
goat anti-mouse biotin	abcam, Cambridge, United Kingdom	ab5886	IHC: 1:100

goat anti-rat HRP	Sigma Aldrich, St. Louis, USA	A9037	WB: 1:1000
goat anti-rabbit biotin	abcam, Cambridge, United Kingdom	ab6012	IHC: 1:100
other			
anti-human Fab fragments	Jackson ImmunoResearch, West Grove, USA	109-007-003	IF (meiotic spreads): 50 µg / mL
IgG goat	Merck, Darmstadt, Germany	I5256	adapted to primary antibody
IgG mouse	Merck, Darmstadt, Germany	I5381	adapted to primary antibody
IgG rabbit	Merck, Darmstadt, Germany	I5006	adapted to primary antibody

abbreviations: IHC: immunohistochemistry, IF: immunofluorescence, WB: Western blot

Appendix Table S4. ClinVar accession numbers of the variants in *M1AP, SHOC1, TEX11,* and *SPO16*

ClinVar Accession number	Gene	Transcript	Variant
SCV001146779.2	*M1AP*	NM_138804.4	c.676dup
SCV004708228	*M1AP*	NM_138804.4	c.1073_1074+10del
SCV004708229	*TEX11*	NM_001003811.2	c.1052dup
SCV004708230	*TEX11*	NM_001003811.2	c.(-157_-99+1)_(738-1_792+1)del
SCV004708231	*TEX11*	NM_001003811.2	c.1245G>A
SCV004708232	*TEX11*	NM_001003811.2	c.(652-1_737+1)_(738-1_792+1)del
SCV004708233	*TEX11*	NM_001003811.2	c.731G>A
SCV004708239.1	*TEX11*	NM_001003811.2	c.1837+1G>C
SCV004708240.1	*TEX11*	NM_001003811.2	c.22del
SCV004708241.1	*TEX11*	NM_001003811.2	c.792+1G>A
SCV004708242.1	*TEX11*	NM_001003811.2	c.(204+1_205-1)_(737+1_738-1)del
SCV004708234	*C1orf146*	NM_001012425.2	c.266del
SCV004708235	*SHOC1*	NM_173521.5	c.1939+2T>C
SCV004708236	*SHOC1*	NM_173521.5	c.1351del
SCV004708237	*SHOC1*	NM_173521.5	c.1347T>A
SCV004708238	*SHOC1*	NM_173521.5	c.945_948del
SCV004708239	*TEX11*	NM_001003811.2	c.1837+1G>C
SCV004708240	*TEX11*	NM_001003811.2	c.22del
SCV004708241	*TEX11*	NM_001003811.2	c.792+1G>A
SCV004708242	*TEX11*	NM_001003811.2	c.(159+1_160-1)_(692+1_693-1)del

The gene symbol *C1orf146* corresponds to its alias *SPO16*.

Appendix Figures

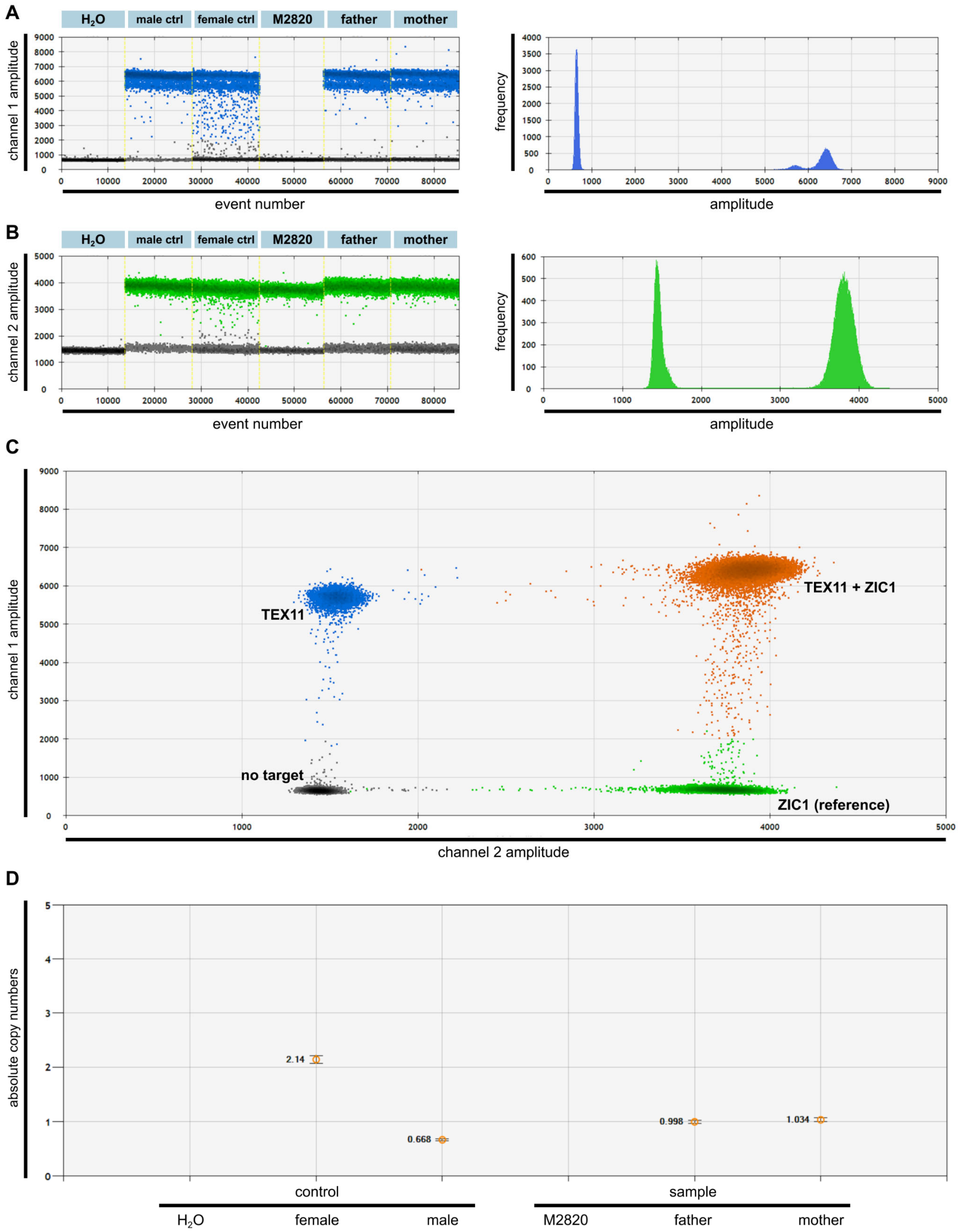

Appendix Figure S1. Digital droplet PCR confirmed the deletion of TEX11 exons 1-11 in M2820. A./B. The 1D-plot showed a clear division of positive droplets (blue and green bands/peaks) and negative droplets (grey bands/peaks), which is an important quality parameter. Depicted are the *TEX11* (A) and a reference (ZIC1, B) measurements. C. The 2D-plot with four distinct droplet groups (6-FAM-positive droplets (blue), HEX-positive droplets (green), double positive droplets (orange) and negative droplets (grey)) confirmed the probe specificity. D. Calculated copy numbers showed the deletion of the respective region of *TEX11* in M2820. The H_2O control did not show any fluorescent droplets, as expected. The female control shows a copy number of two and the male control shows a copy number of one, as *TEX11* is a X-chromosomal gene. M2820's father carries one copy, as expected. However, his mother only shows one copy and therefore is a heterozygous carrier of the deletion.

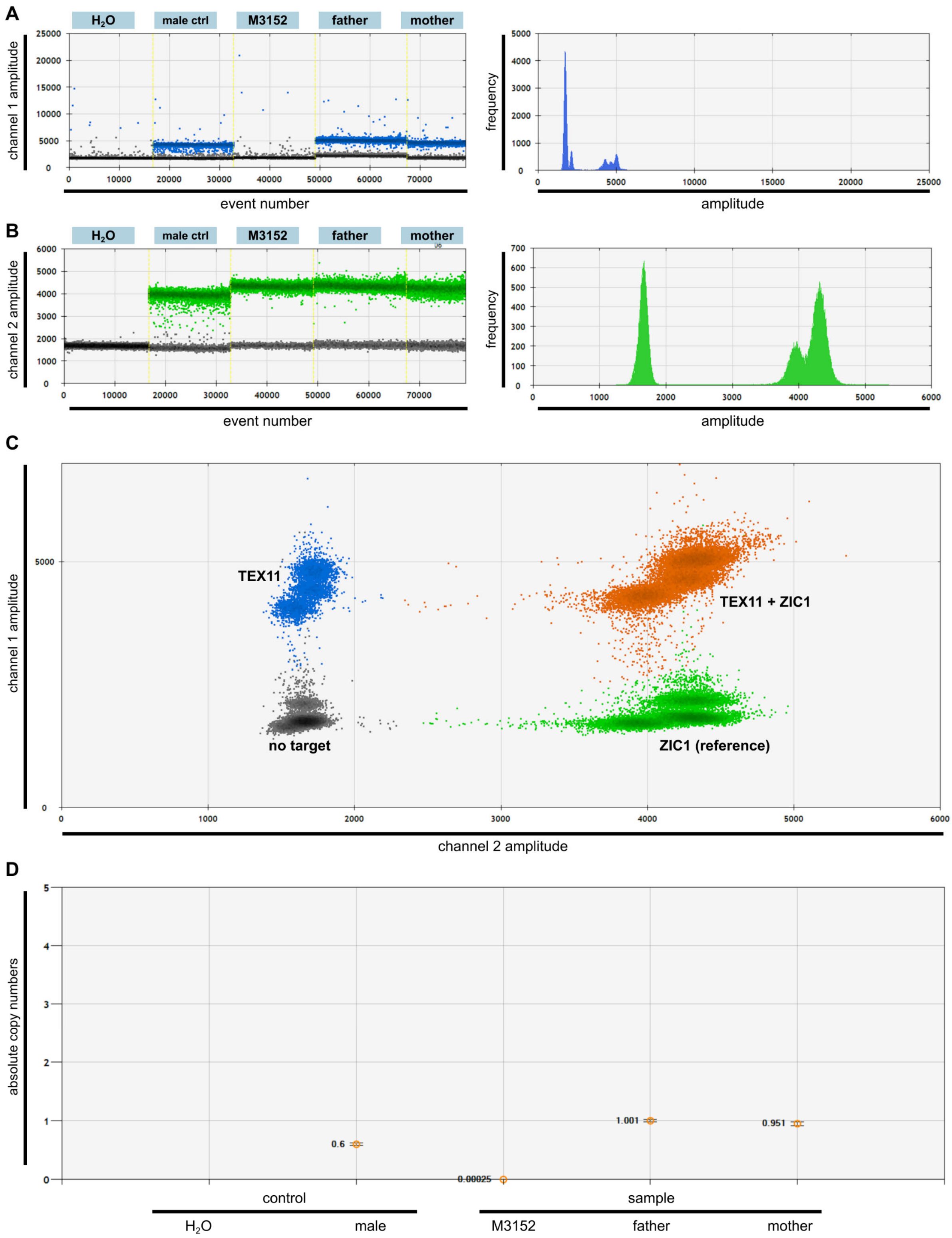

Appendix Figure S2. Digital droplet PCR confirms the deletion of TEX11 exons 10-11 in M3152. A./B. Positive and negative droplets were clearly distinguishable in the *TEX11* and reference measurement (ZIC1). C. Quality parameter confirmed probe specificity. D. Calculated copy numbers showed the deletion of the respective region of *TEX11* in M3152. The H_2O control did not show any fluorescent droplets and the male control showed a copy number of one, as expected for a X-chromosomal gene. M3152's father carried one copy, as expected. However, his mother only showed one copy and therefore is a heterozygous carrier of the deletion.

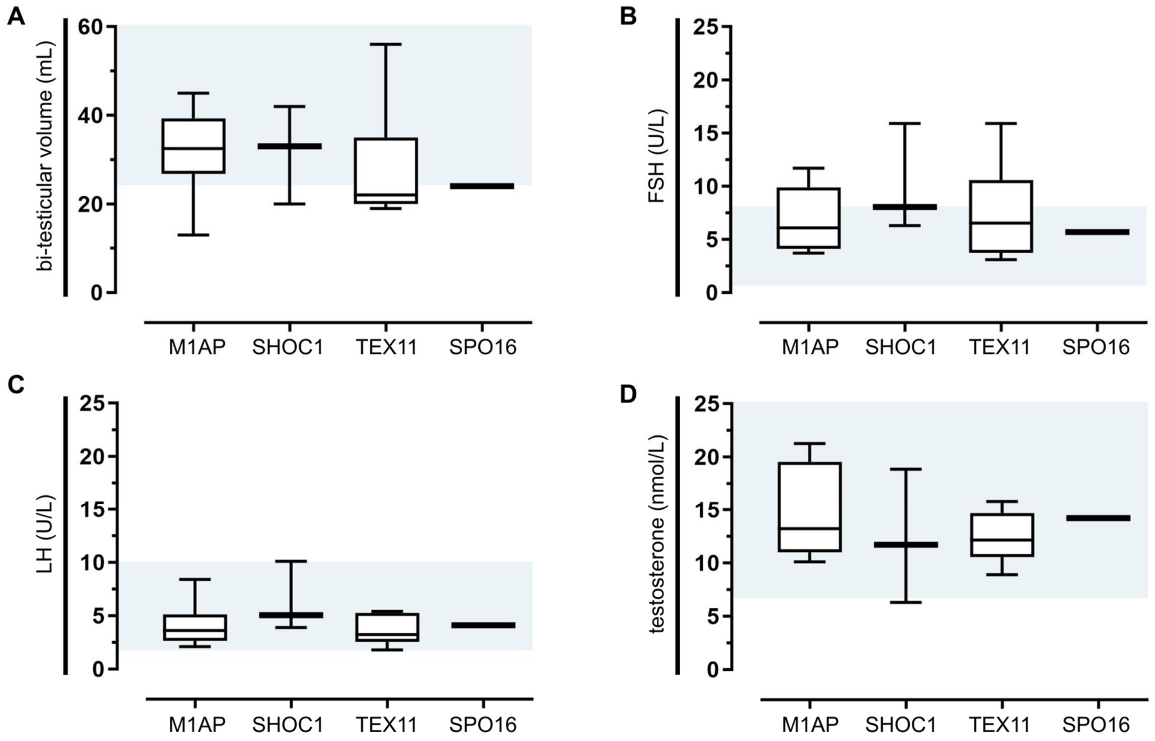

Appendix Figure S3. Overview of clinical data of men with loss-of-function variants in *M1AP*, *SHOC1*, *TEX11* or *SPO16*.
Exome data of men with variants in *M1AP* (N=10), *SHOC1* (N=4), *TEX11* (N=9), and *SPO16* (N=1) was queried and depicted data shows the median values with the respective 95% confidence intervals. A. Bi-testicular volume (mL). B. Serum FSH (U/L). C. Serum LH (U/L). D. Serum testosterone (nmol/L). Blue areas represent respective reference values (FSH = 1-7 IU/L, LH = 2-10 IU/L, T ≥ 12 nmol/L, bi-testicular volume ≥ 24 mL per testis.)

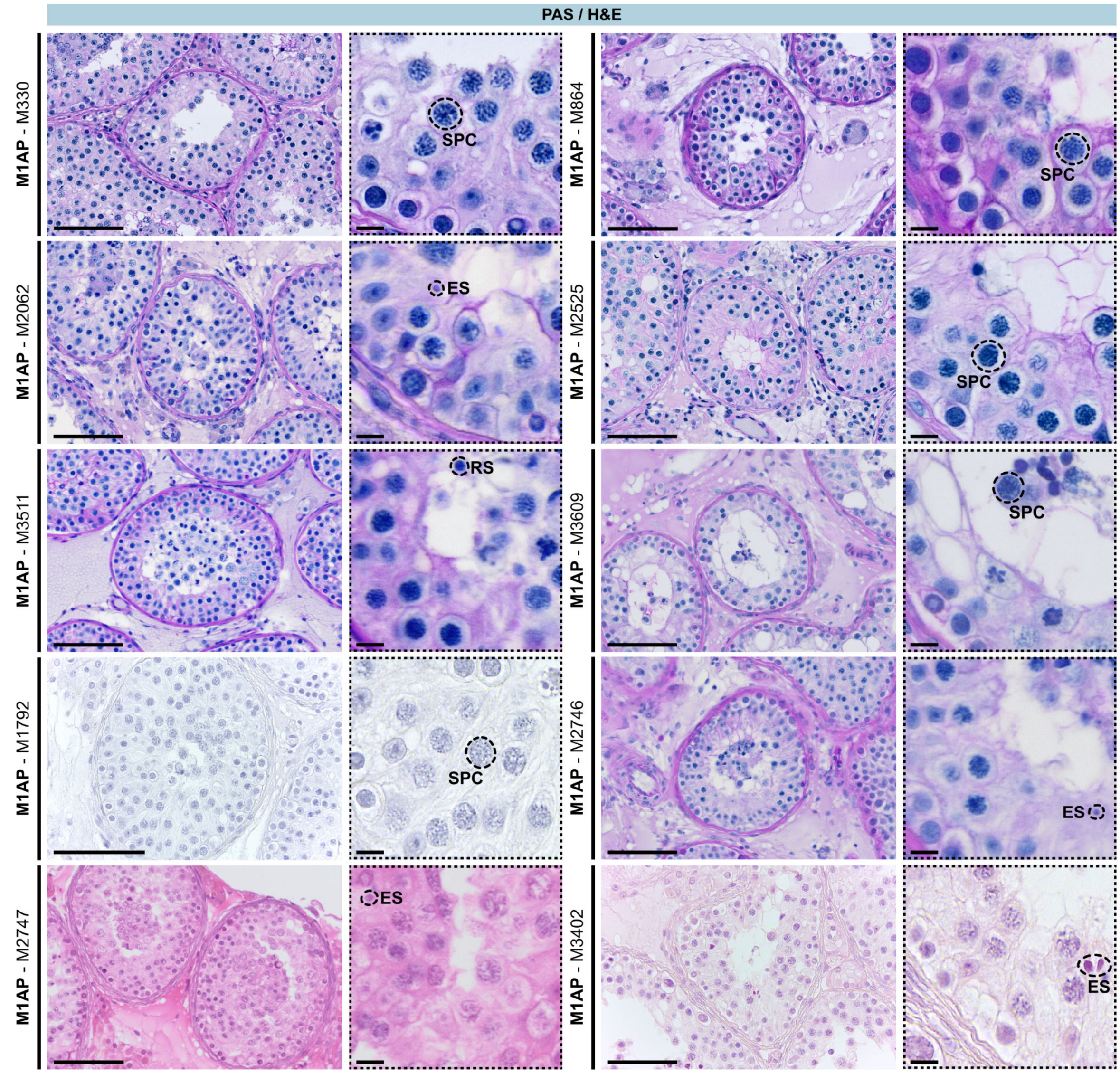

Appendix Figure S4. PAS (N=7) or H&E (N=3) staining of men with LoF variants in *M1AP*.
All men carrying variants in *M1AP* underwent attempts for testicular sperm retrieval (TESE, N=10). Overview staining revealed testicular architecture and germ cell types were quantified for each tubule. SPC = spermatocyte, RS = round spermatid, ES = elongated spermatid. The scale bar represents 100 µm and 10 µm, respectively.

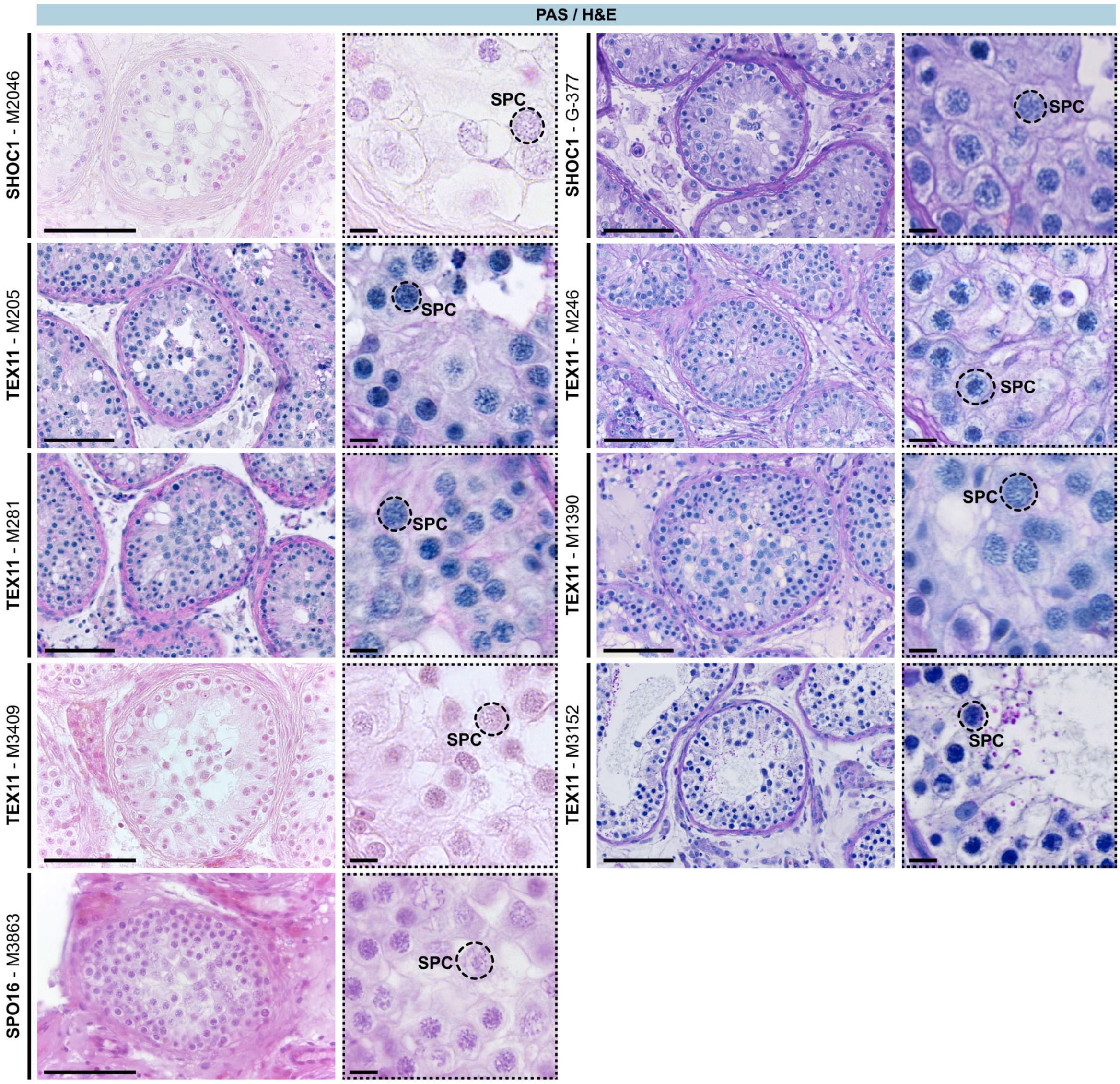

Appendix Figure S5. PAS (N=6) or H&E (N=3) staining of men with LoF variants in *SHOC1*, *TEX11* or *SPO16*. Ten of 14 men carrying variants in *SHOC1*, *TEX11* or *SPO16* underwent testicular surgery for TESE attempt. Overview staining revealed the testicular architecture and germ cell types were quantified for each tubule. SPC = spermatocyte. The scale bar represents 100 µm and 10 µm, respectively.

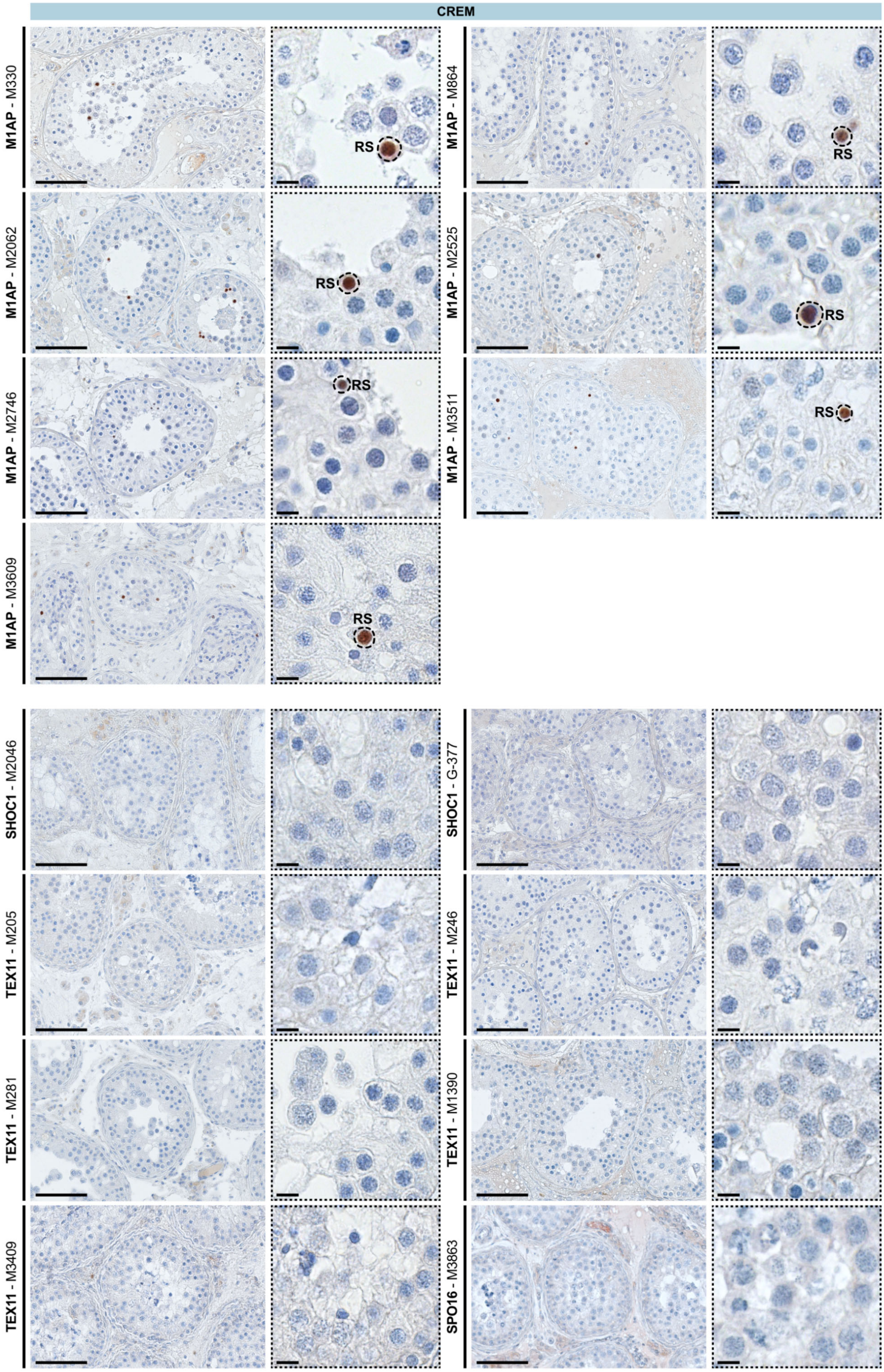

Appendix Figure S6. CREM staining in men with loss-of-function variants in *M1AP*, *SHOC1*, *TEX11* or *SPO16*.

Testicular tissue was stained for CREM to analyse development of haploid round spermatids (RS). Positive cells are indicated in the magnification. The magnification of M1AP – M2746 is also shown in Figure 2B. The scale bar represents 100 µm and 10 µm, respectively.

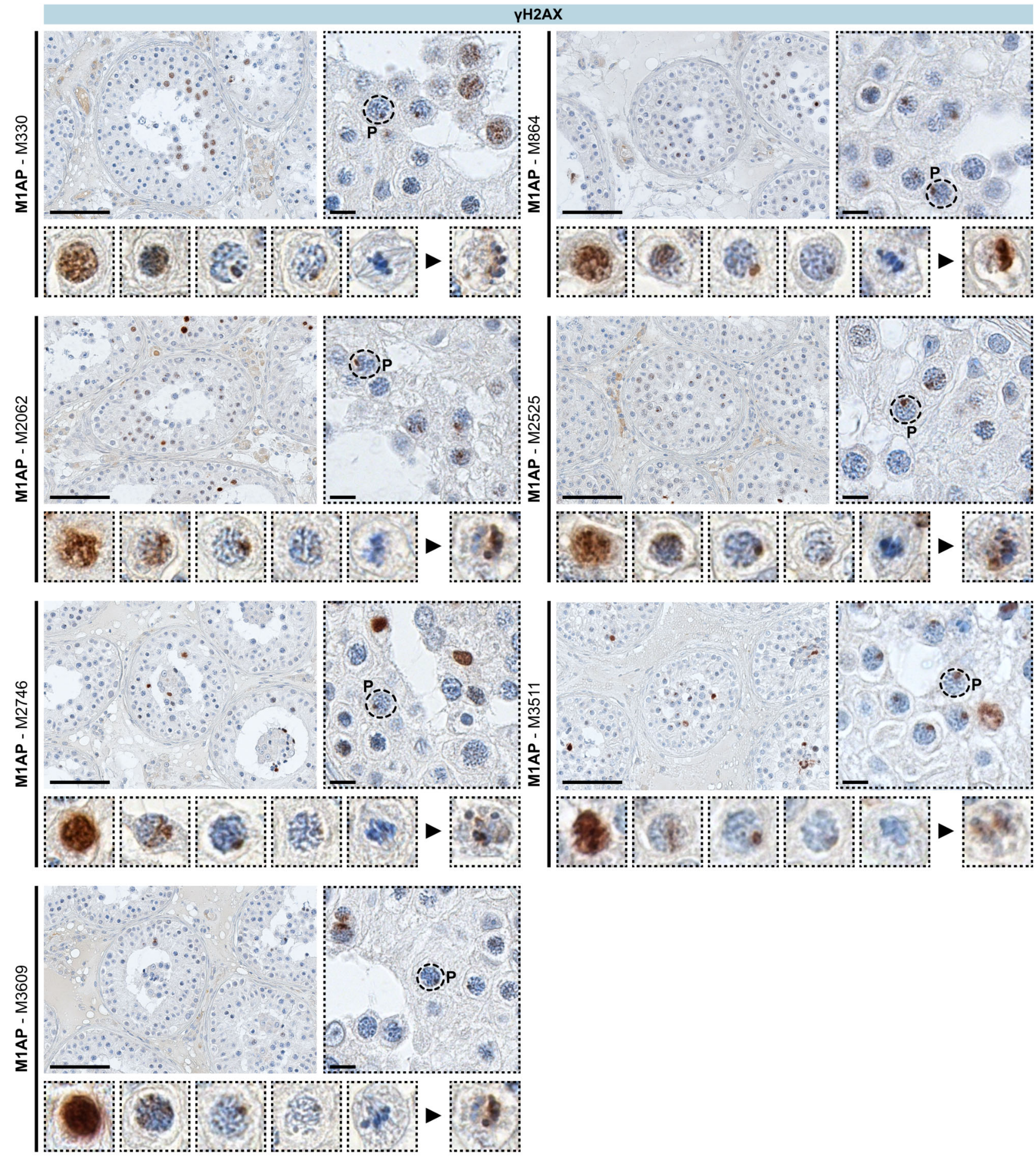

Appendix Figure S7. γH2AX localisation showed meiosis prophase I progression in men with loss-of-function variants in *M1AP*.
Testicular tissue was stained for the DSB marker γH2AX. Meiotic prophase I substages (L = leptotene-, Z = zygotene-, P = pachytene-, D = diplotene-like) and metaphase I (M-I)-like were identified and depicted in the detail view for each man. Besides, aberrant, γH2AX-positive metaphase-like cells are shown (black arrow head). Pachytene-like cells are indicated in the magnification. The scale bar represents 100 µm and 10 µm, respectively.

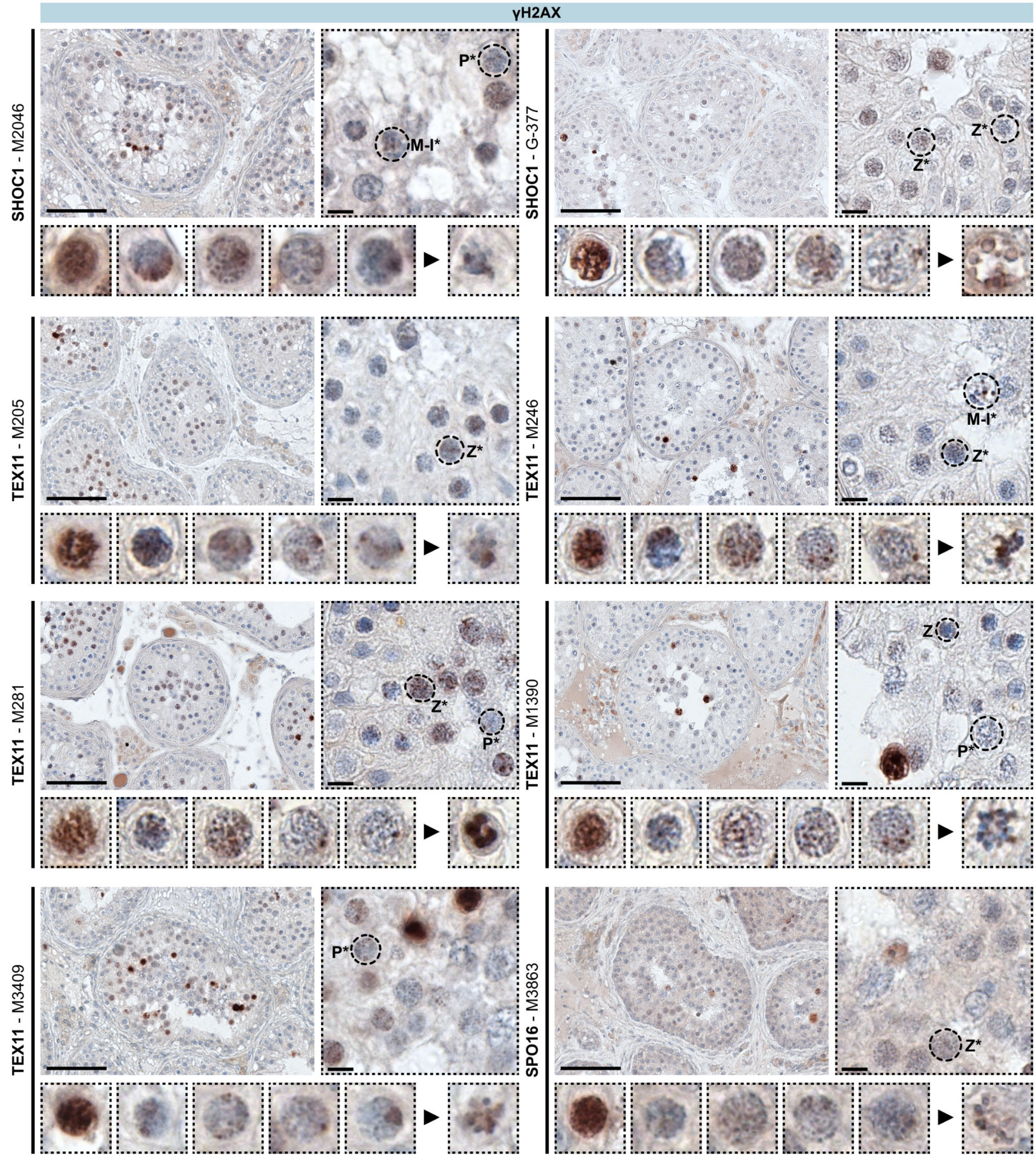

Appendix Figure S8. γH2AX localisation showed impaired meiosis prophase I progression in men with loss-of-function variants in *SHOC1*, *TEX11* or *SPO16*.
Testicular tissue was stained for the DSB marker γH2AX. In M2046 and G-377, the majority of cells reached only a zygotene-like stage. Contrary to the other men with LoF variants in *TEX11*, one man with a frameshift variant in *TEX11* (M3409) had few pachytene-like cells in two of 86 counted seminiferous tubules. These cells had an enlarged XY body-like structure with accumulated γH2AX. Meiotic prophase I substages (L = leptotene-, Z = zygotene-like) and arrested zygotene- (Z*), pachytene- (P*) and metaphase I-like cells (M-I*) were identified and depicted in a detail view. The scale bar represents 100 µm and 10 µm, respectively.

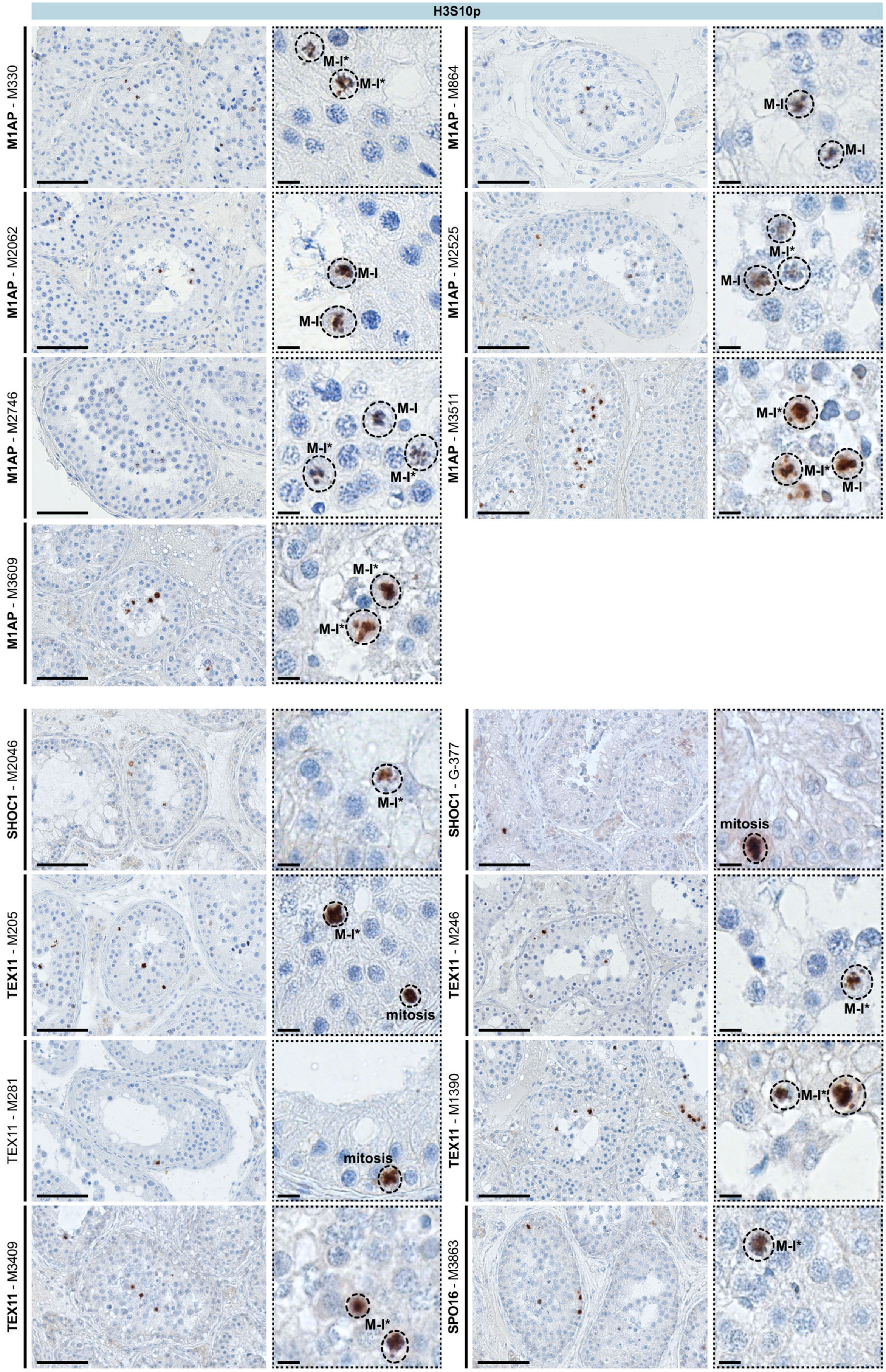

112
113 **Appendix Figure S9. Metaphase I cells were determined by H3S10p localisation in men with loss-of-function**
114 **variants in *M1AP*, *SHOC1*, *TEX11* or *SPO16*.**

Testicular tissue was stained for H3S10p to detect diakinesis / aberrant metaphase-I-like (M-I*) or normal metaphase I-like (M-I) cells. Localisation of positive cells was taken into account to distinguish between mitotic (spermatogonia = mitosis) and meiotic (spermatocyte) M-I cells (indicated in magnification). The scale bar represents 100 µm and 10 µm, respectively.

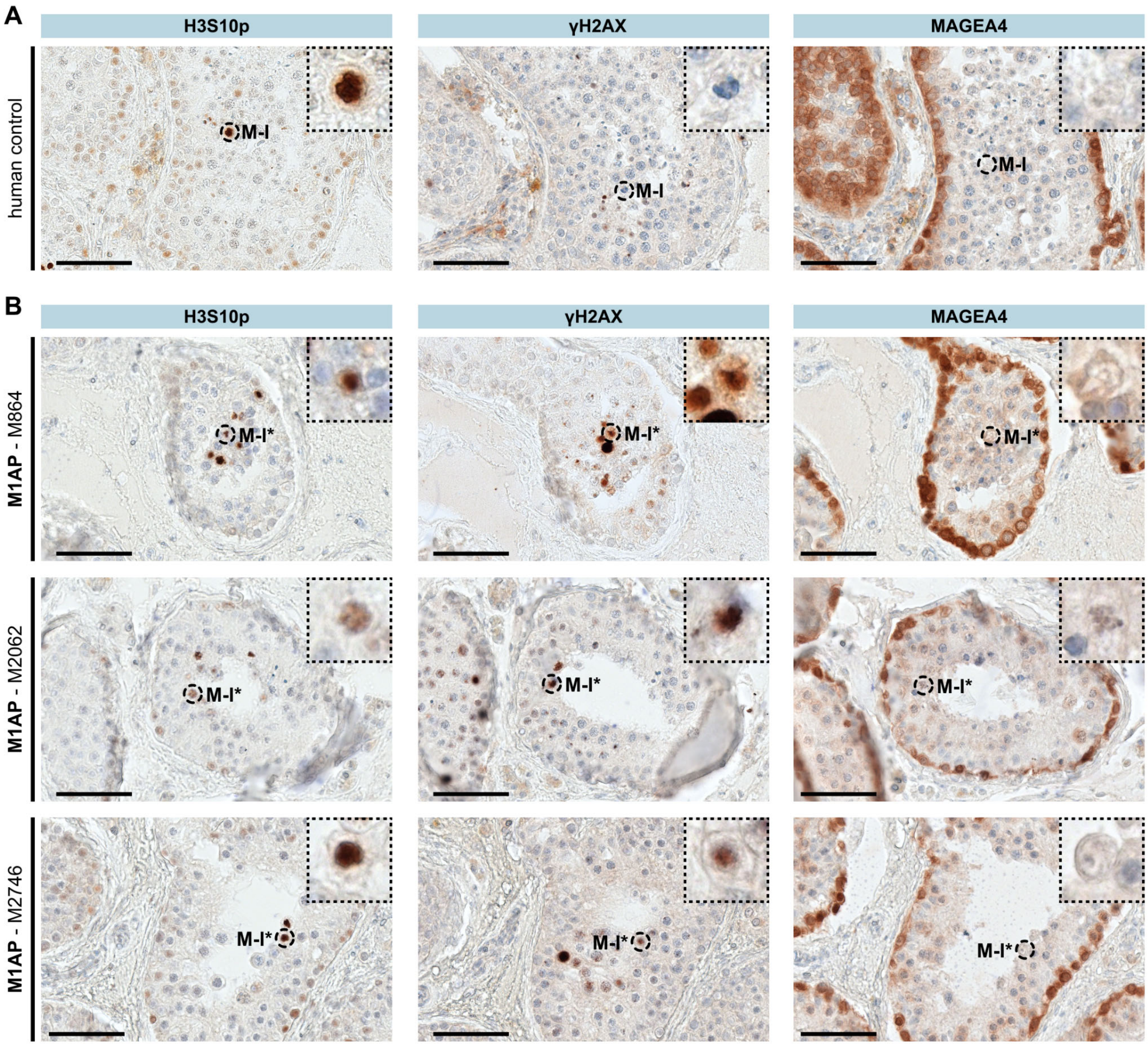

Appendix Figure S10. Immunohistological staining of H3S10p, γH2AX, and MAGEA4 of sequential testicular sections with a distance of 3 µm.

IHC was performed to confirm that metaphase-I (M-I) and metaphase-I like (M-I*) cells were spermatocytes and not spermatogonia. The scale bar represents 100 µm.

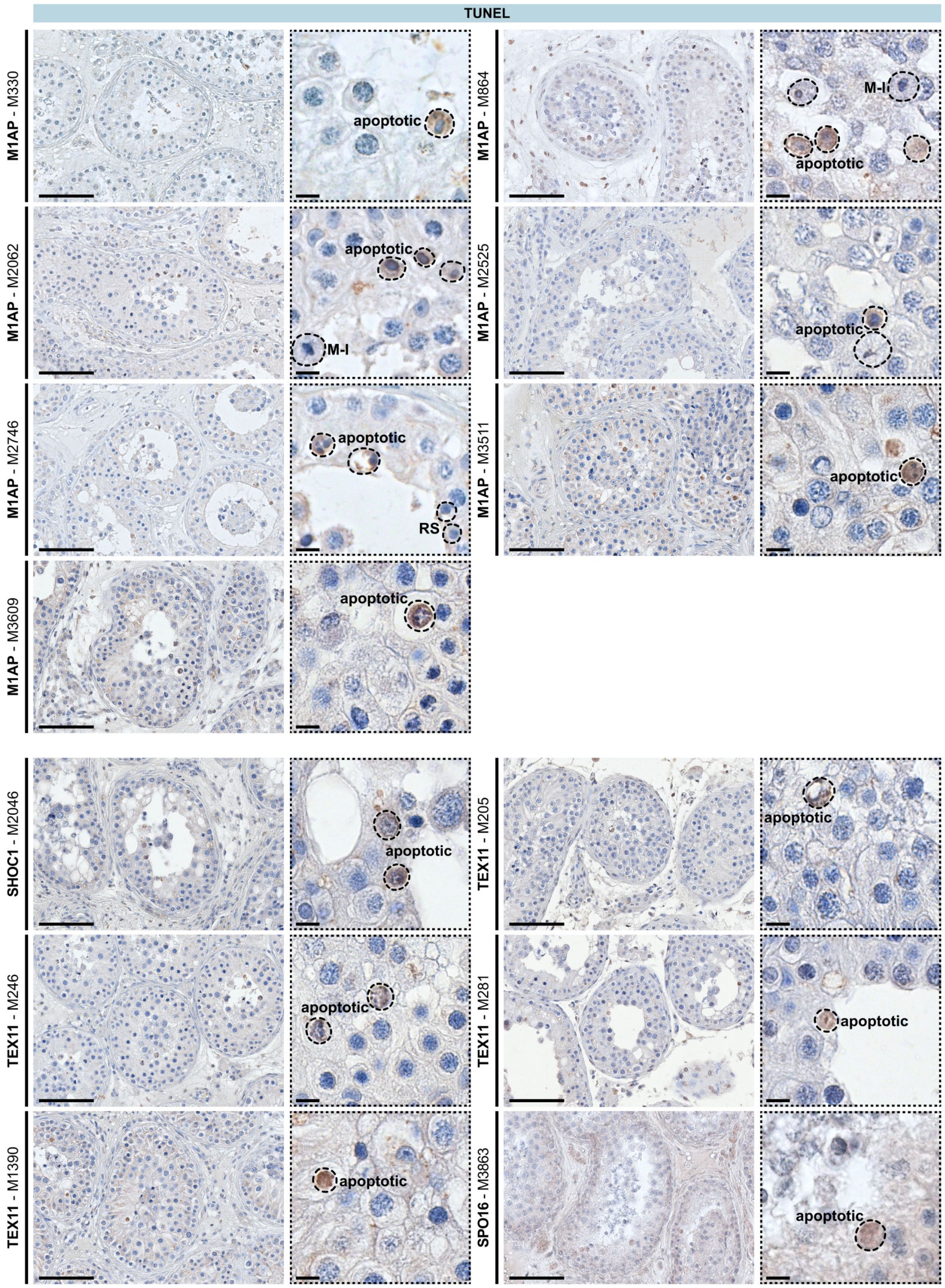

Appendix Figure S11. Germ cell apoptosis in men with loss-of-function variants in *M1AP*, *SHOC1*, *TEX11* or *SPO16*.
Testicular tissue was analysed by TUNEL assay to show apoptotic cells. Positive apoptotic cells, negative diakinesis / metaphase-I-like(M-I) cells, and negative round spermatids (RS) are indicated in the magnification. The scale bar represents 100 µm and 10 µm, respectively.

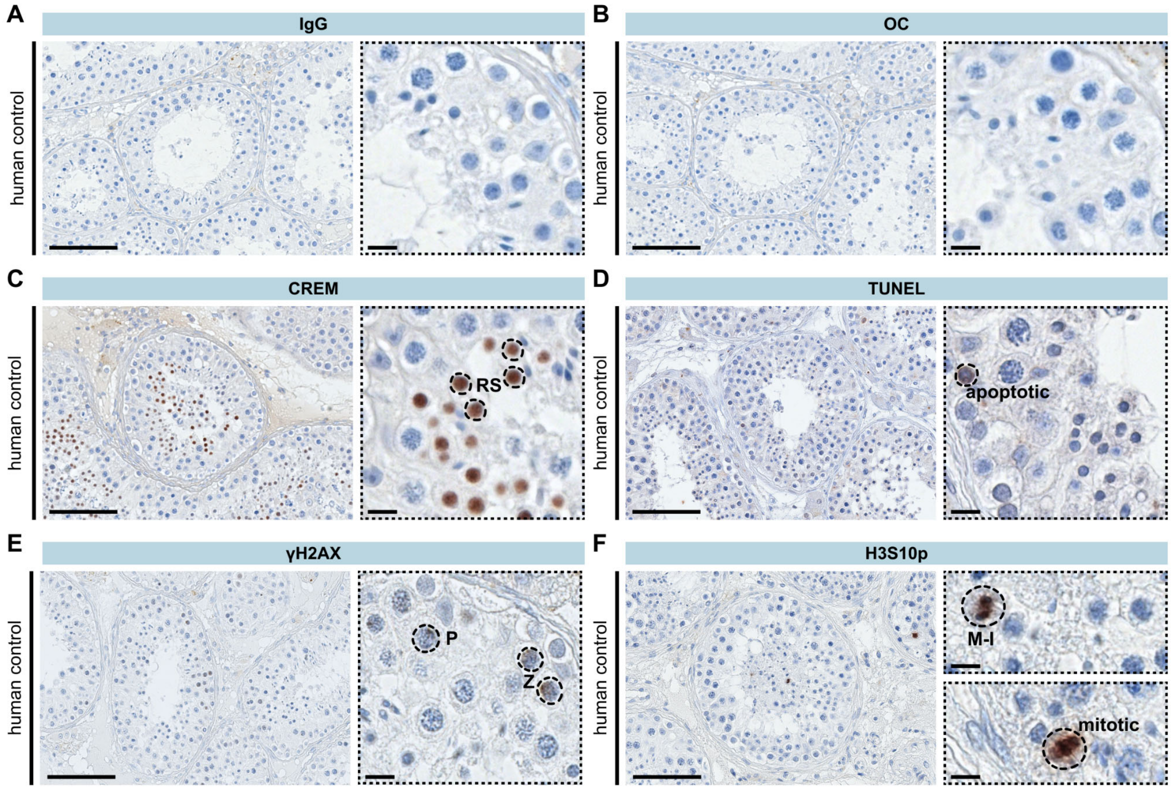

Appendix Figure S12. Staining of testicular tissue from a representative human control.
A. Isotype control (IgG). B. Omission of first antibody control (OC). C. CREM staining. D. TUNEL assay. E. γH2AX staining. F. H3S10p staining. Positive cells are indicated in the magnification. The scale bar represents 100 µm and 10 µm.

Appendix Reference

An M, Liu Y, Zhang M, Hu K, Jin Y, Xu S, Wang H & Lu M (2021) Targeted next-generation sequencing panel screening of 668 Chinese patients with non-obstructive azoospermia. *J Assist Reprod Genet* 38: 1997–2005

Chen S, Wang G, Zheng X, Ge S, Dai Y, Ping P, Chen X, Liu G, Zhang J, Yang Y, *et al* (2020) Whole-exome sequencing of a large Chinese azoospermia and severe oligospermia cohort identifies novel infertility causative variants and genes. *Hum Mol Genet* 29: 2451–2459

Ji Z, Yao C, Yang C, Huang C, Zhao L, Han X, Zhu Z, Zhi E, Liu N, Zhou Z, *et al* (2021) Novel Hemizygous Mutations of TEX11 Cause Meiotic Arrest and Non-obstructive Azoospermia in Chinese Han Population. *Front Genet* 12: 1–10

Khan MR, Akbari A, Nicholas TJ, Castillo-Madeen H, Ajmal M, Haq TU, Laan M, Quinlan AR, Ahuja JS, Shah AA, *et al* (2023) Genome sequencing of Pakistani families with male infertility identifies deleterious genotypes in SPAG6, CCDC9, TKTL1, TUBA3C, and M1AP. *Andrology*: 1–12

Krausz C, Riera-Escamilla A, Moreno-Mendoza D, Holleman K, Cioppi F, Algaba F, Pybus M, Friedrich C, Wyrwoll MJ, Casamonti E, *et al* (2020) Genetic dissection of spermatogenic arrest through exome analysis: clinical implications for the management of azoospermic men. *Genet Med* 22: 1956–1966

Li Y, Wu Y, Khan I, Zhou J, Lu Y, Ye J, Liu J, Xie X, Hu C, Jiang H, *et al* (2023) M1AP interacts with the mammalian ZZS complex and promotes male meiotic recombination. *EMBO Rep* 24: e55778

Nagirnaja L, Lopes AM, Charng WL, Miller B, Stakaitis R, Golubickaite I, Stendahl A, Luan T, Friedrich C, Mahyari E, *et al* (2022) Diverse monogenic subforms of human spermatogenic failure. *Nat Commun* 13: 7953

Song J, Sha Y, Liu X & Zeng X (2023) Novel mutations of TEX11 are associated with non-obstructive azoospermia. 1–8

Tang D, Li K, Geng H, Xu C, Lv M, Gao Y, Wang G, Yu H, Shao Z, Shen Q, *et al* (2022) Identification of deleterious variants in patients with male infertility due to idiopathic non-obstructive azoospermia. *Reprod Biol Endocrinol* 20: 1–11

Tu C, Wang Y, Nie H, Meng L, Wang W, Li Y, Li D, Zhang H, Lu G, Lin G, *et al* (2020) An M1AP homozygous splice-site mutation associated with severe oligozoospermia in a consanguineous family. *Clin Genet* 97: 741–746

Wang W, Meng L, He J, Su L, Li Y, Tan C, Xu X, Nie H, Zhang H, Du J, *et al* (2022) Bi-Allelic variants in SHOC1 cause non-obstructive azoospermia with meiosis arrest in humans and mice. *Mol Hum Reprod* 28: 1–13

Wyrwoll MJ, Köckerling N, Vockel M, Dicke AK, Rotte N, Pohl E, Emich J, Wöste M, Ruckert C, Wabschke R, *et al* (2023) Genetic Architecture of Azoospermia—Time to Advance the Standard of Care. *Eur Urol* 83: 452–462

Wyrwoll MJ, Temel ŞG, Nagirnaja L, Oud MS, Lopes AM, van der Heijden GW, Heald JS, Rotte N, Wistuba J, Wöste M, *et al* (2020) Bi-allelic Mutations in M1AP Are a Frequent Cause of Meiotic Arrest and Severely Impaired Spermatogenesis Leading to Male Infertility. *Am J Hum Genet* 107: 342–351

Yang F, Silber S, Leu NA, Oates RD, Marszalek JD, Skaletsky H, Brown LG, Rozen S, Page DC & Wang PJ (2015) TEX11 is mutated in infertile men with azoospermia and regulates genome-wide recombination rates in mouse. *EMBO Mol Med* 7: 1198–1210

Yao C, Yang C, Zhao L, Li P, Tian R, Chen H, Guo Y, Huang Y, Zhi E, Zhai J, *et al* (2021) Bi-allelic SHOC1 loss-of-function mutations cause meiotic arrest and non-obstructive azoospermia. *J Med Genet* 58: 679–686

Yatsenko AN, Georgiadis AP, Röpke A, Berman AJ, Jaffe T, Olszewska M, Westernströer B, Sanfilippo

J, Kurpisz M, Rajkovic A, *et al* (2015) X-Linked TEX11 Mutations, Meiotic Arrest, and Azoospermia in Infertile Men. *N Engl J Med* 372: 2097–2107

Yu XC, Li MJ, Cai FF, Yang SJ, Liu H Bin & Zhang HB (2021) A new TEX11 mutation causes azoospermia and testicular meiotic arrest. *Asian J Androl* 23: 510–515
