# Supplementary figures and images for: Genotype-specific differences in infertile men due to loss-of-function variants in M1AP or ZZS genes
Source: EMBO Mol Med. 2025 May 15;17(6):1417–51. doi: 10.1038/s44321-025-00244-0 (PMC12162868; doi:10.1038/s44321-025-00244-0)

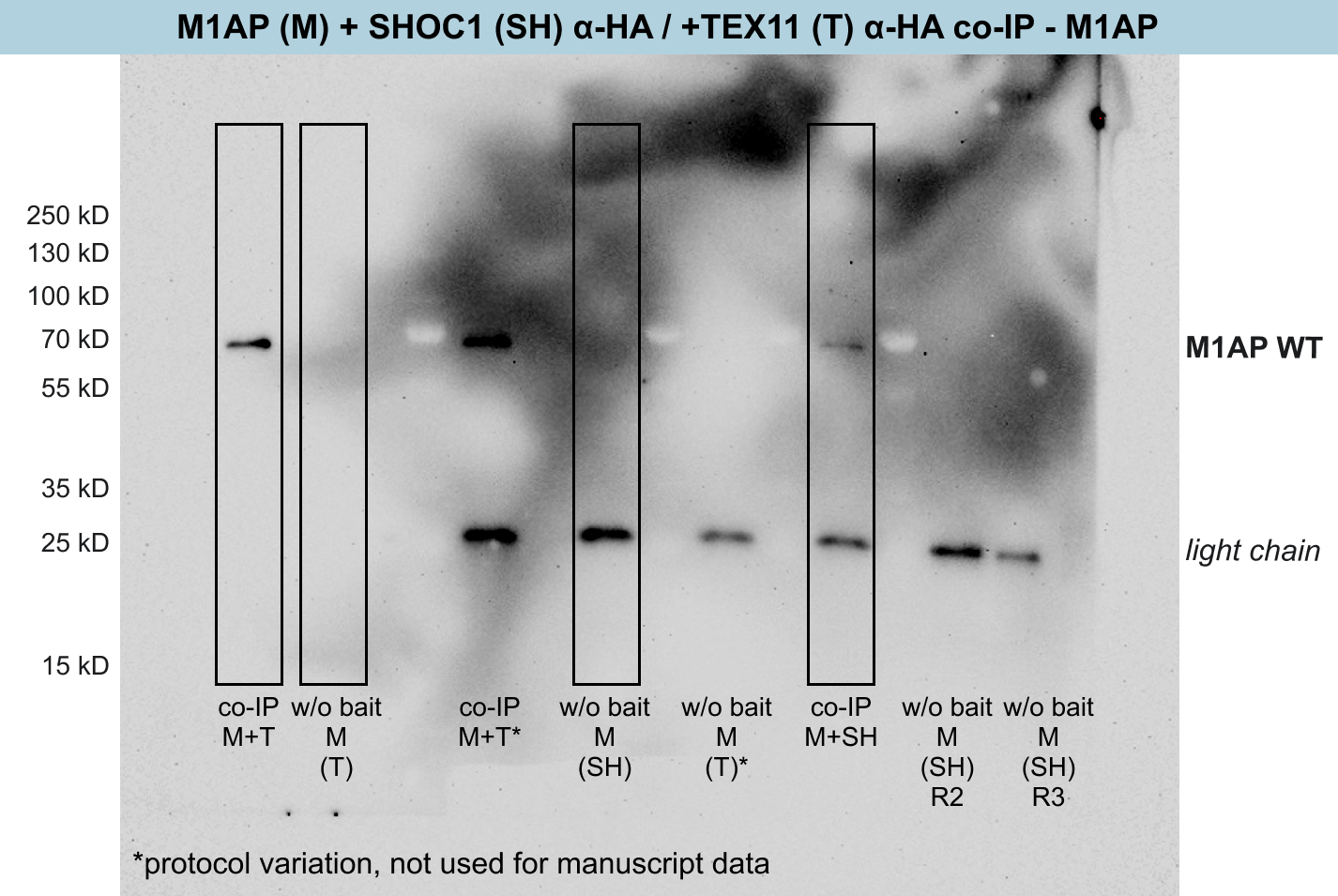

Supplement: Supplementary file 4 — Source data Fig. 1 [file 44321_2025_244_MOESM4_ESM.zip › Source Data_Figure 1_Blots/Figure 1B Western blot_M1AP-co-IP-A-B.tif]

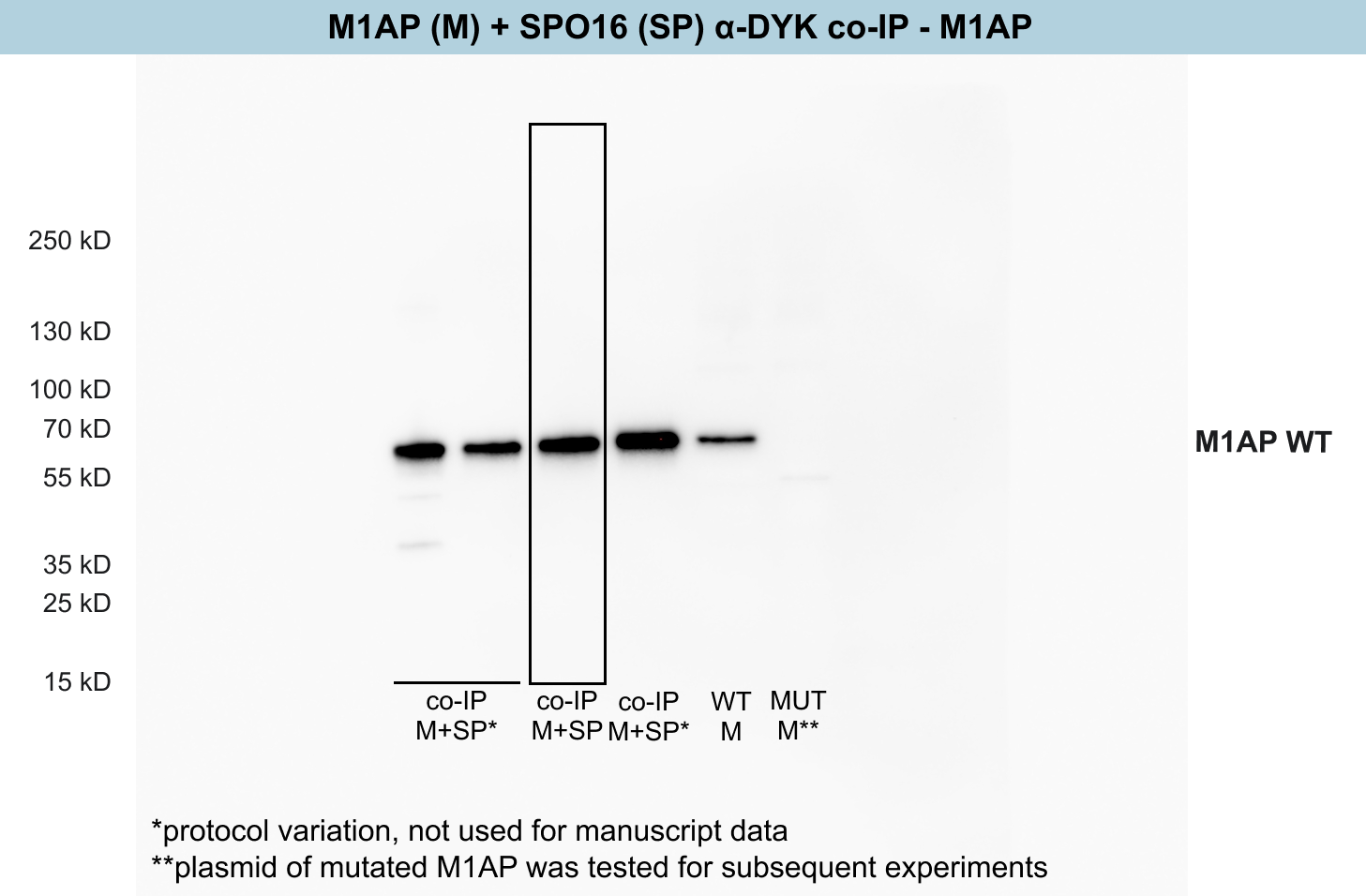

Supplement: Supplementary file 4 — Source data Fig. 1 [file 44321_2025_244_MOESM4_ESM.zip › Source Data_Figure 1_Blots/Figure 1B Western blot_M1AP-co-IP-C.tif]

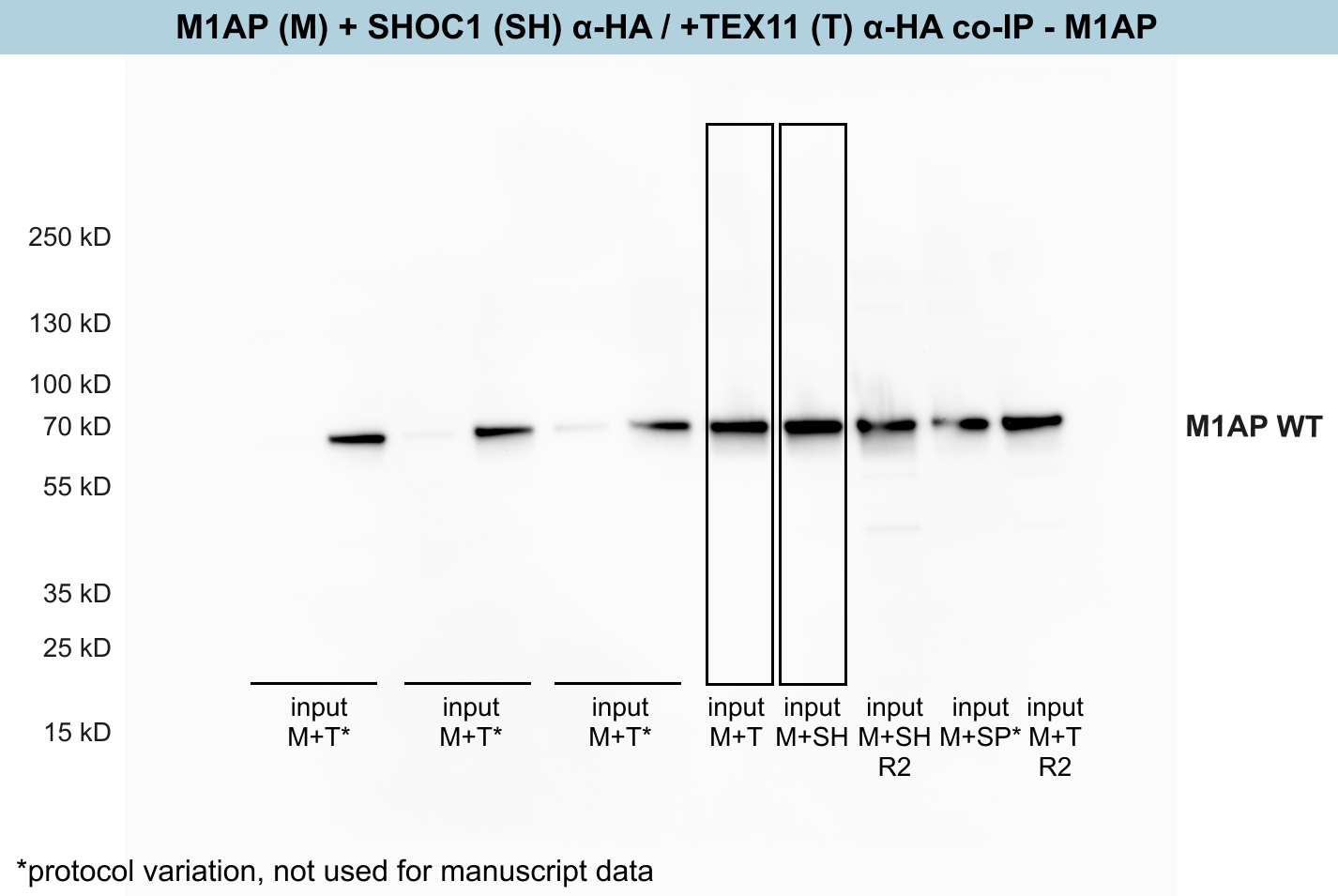

Supplement: Supplementary file 4 — Source data Fig. 1 [file 44321_2025_244_MOESM4_ESM.zip › Source Data_Figure 1_Blots/Figure 1B Western blot_M1AP-input-A-B.tif]

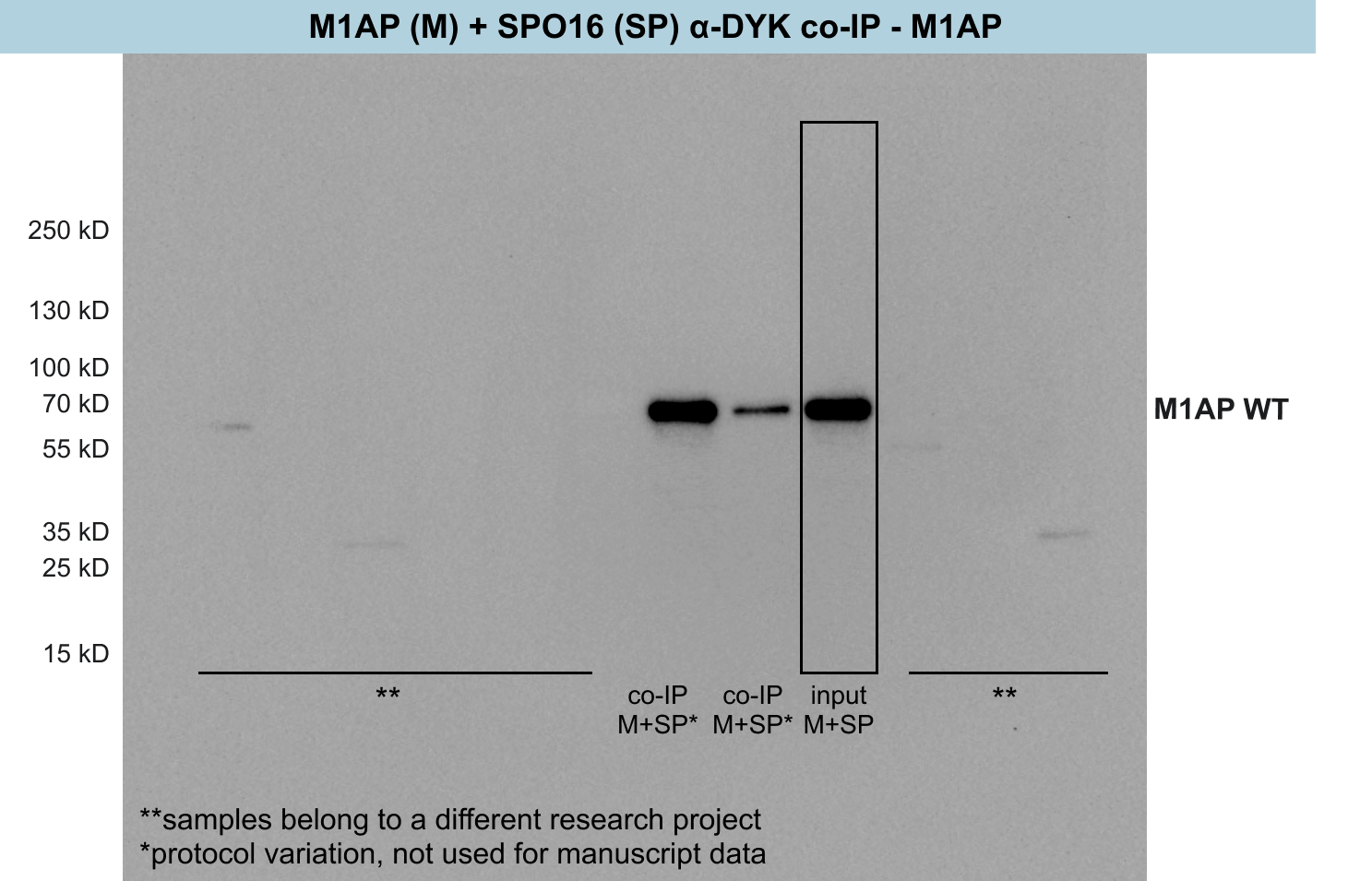

Supplement: Supplementary file 4 — Source data Fig. 1 [file 44321_2025_244_MOESM4_ESM.zip › Source Data_Figure 1_Blots/Figure 1B Western blot_M1AP-input-C.tif]

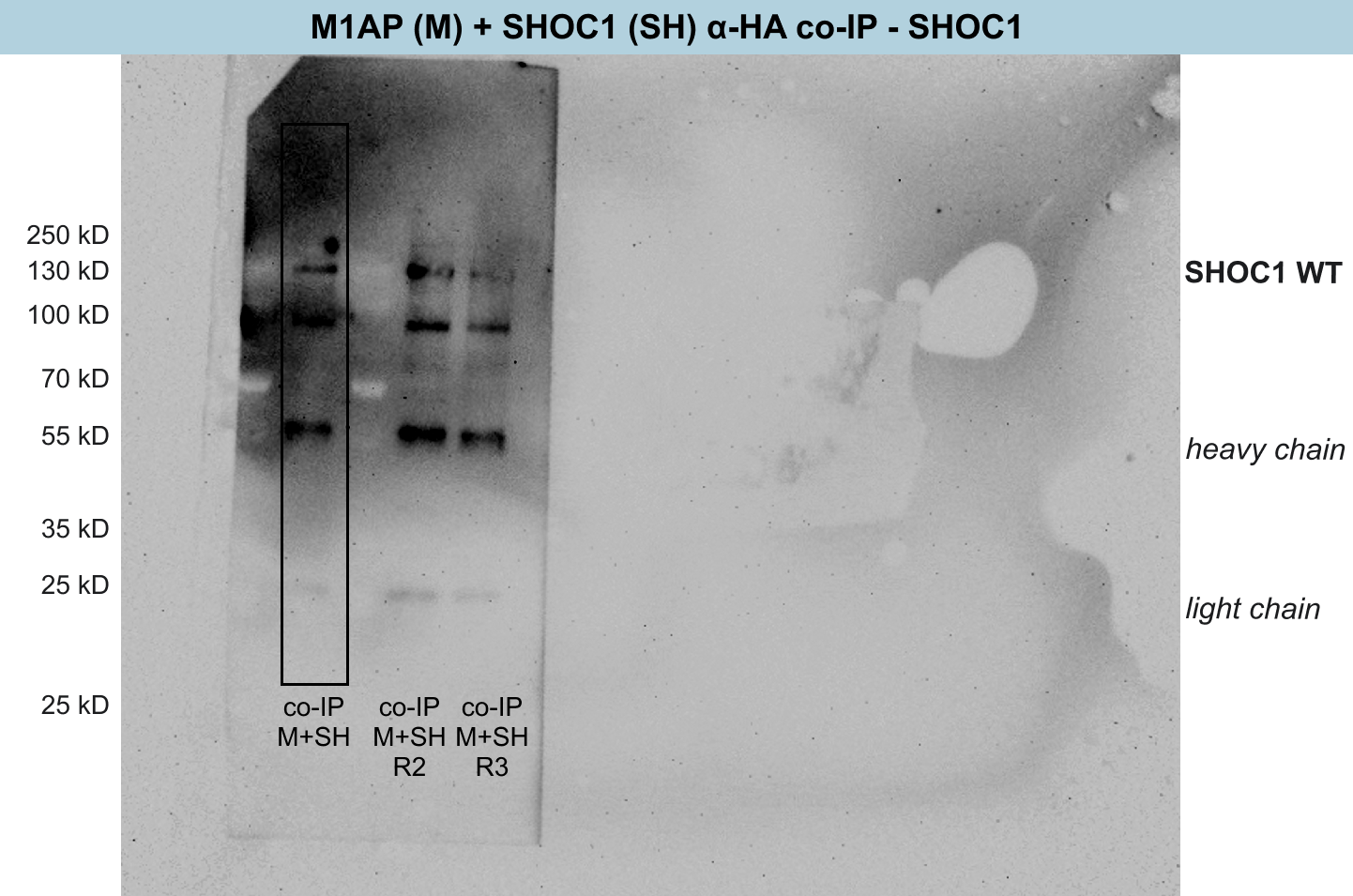

Supplement: Supplementary file 4 — Source data Fig. 1 [file 44321_2025_244_MOESM4_ESM.zip › Source Data_Figure 1_Blots/Figure 1B Western blot_SHOC1-co-IP.tif]

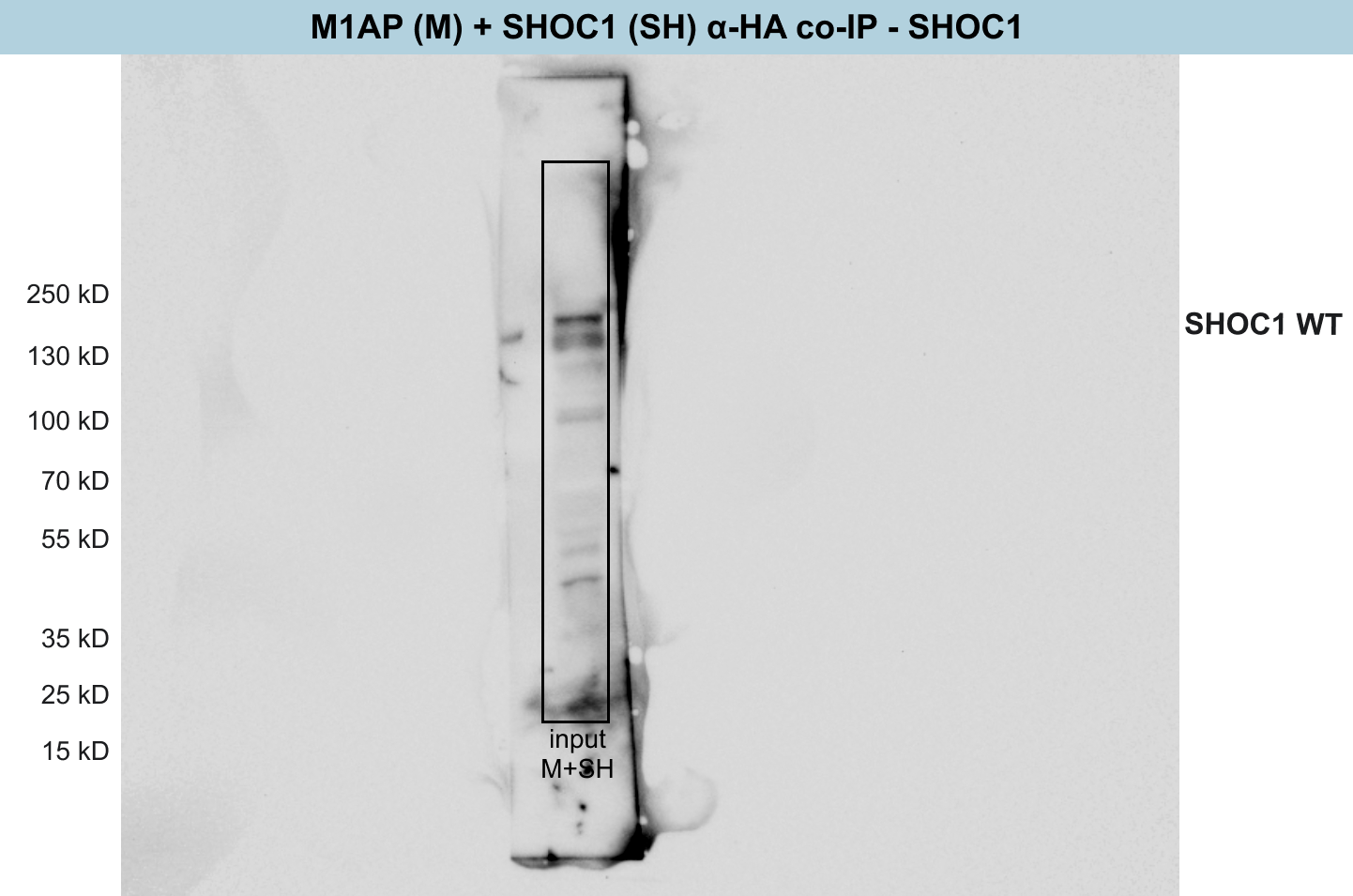

Supplement: Supplementary file 4 — Source data Fig. 1 [file 44321_2025_244_MOESM4_ESM.zip › Source Data_Figure 1_Blots/Figure 1B Western blot_SHOC1-input.tif]

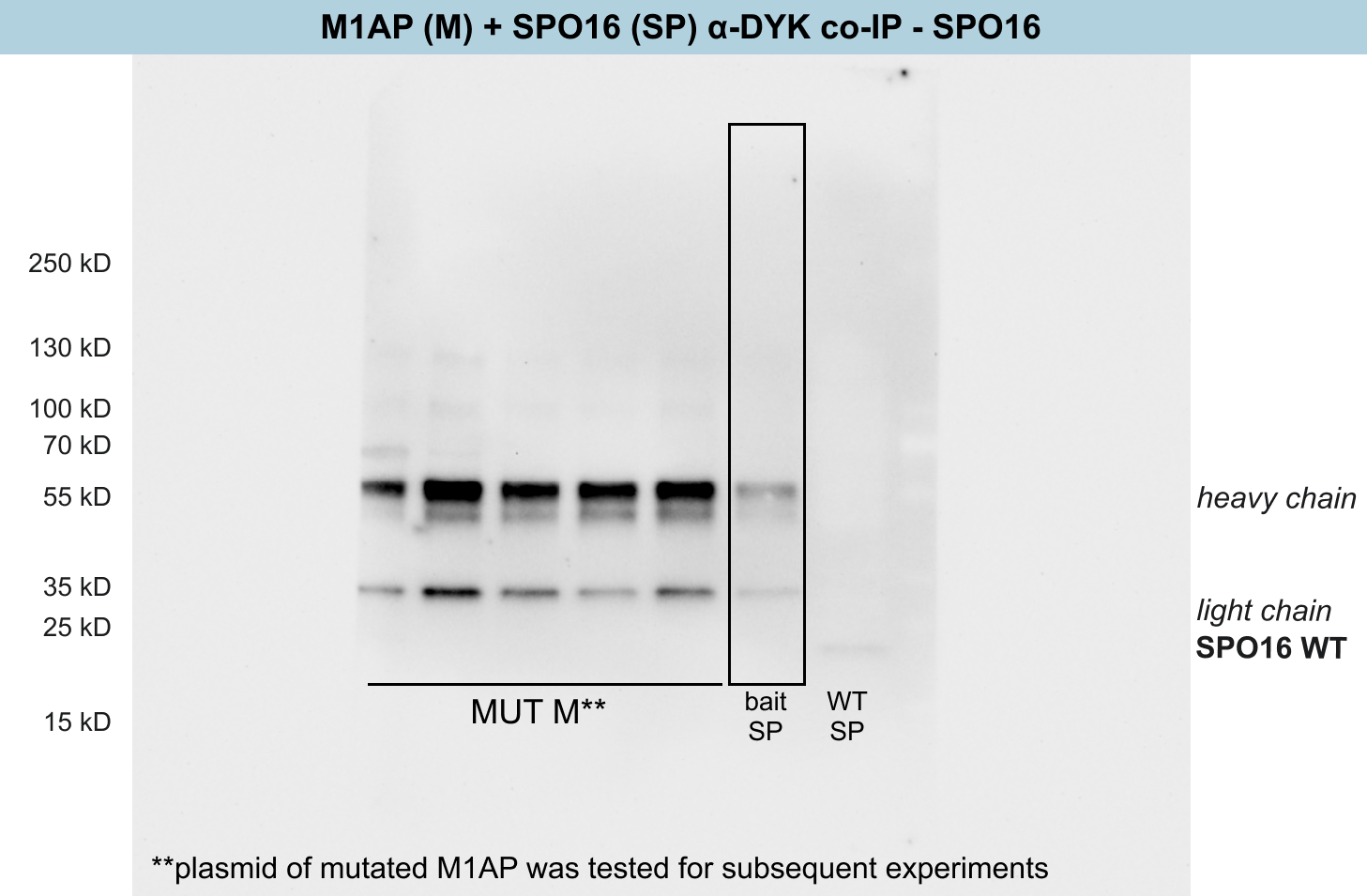

Supplement: Supplementary file 4 — Source data Fig. 1 [file 44321_2025_244_MOESM4_ESM.zip › Source Data_Figure 1_Blots/Figure 1B Western blot_SPO16-co-IP.tif]

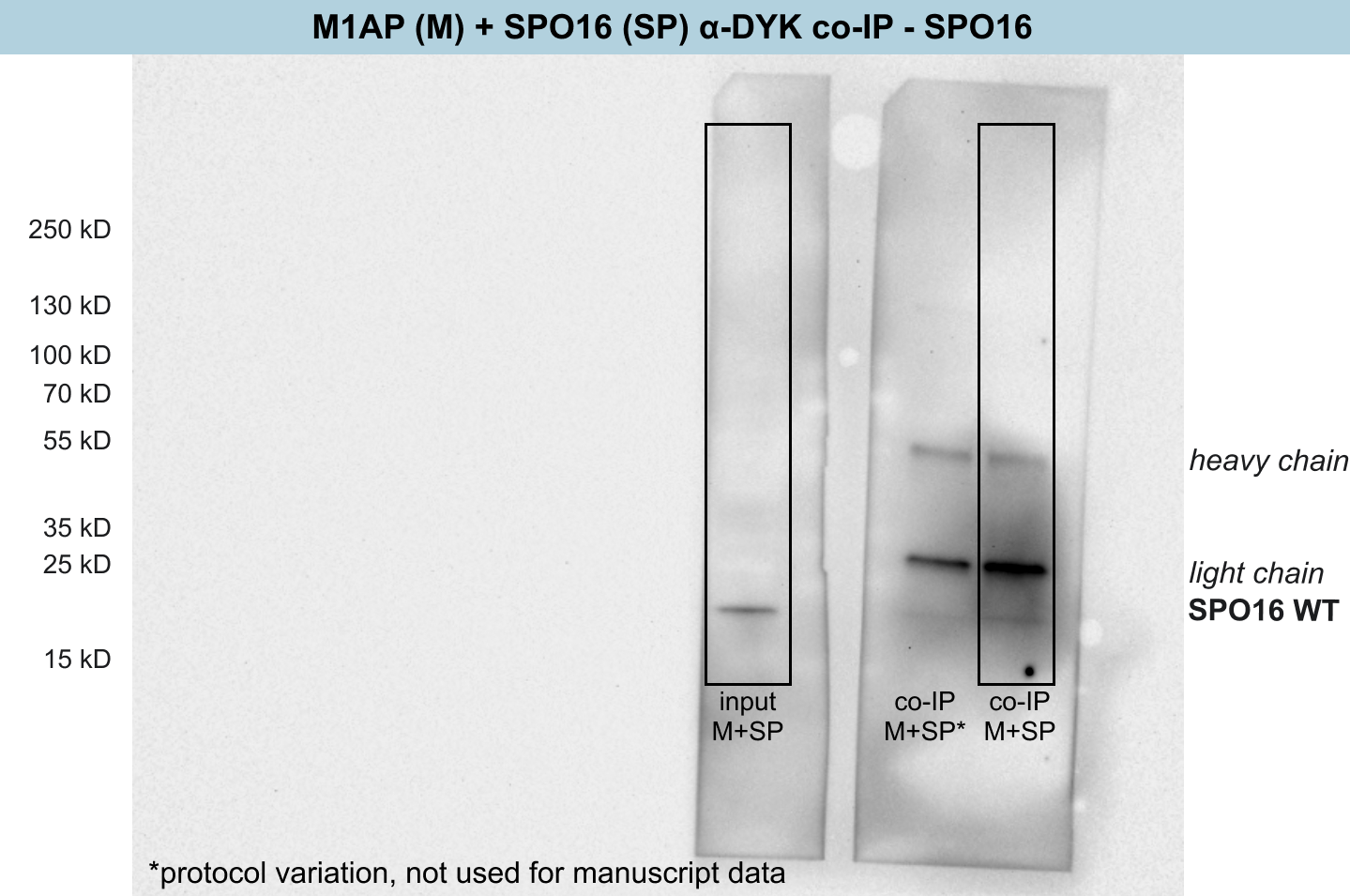

Supplement: Supplementary file 4 — Source data Fig. 1 [file 44321_2025_244_MOESM4_ESM.zip › Source Data_Figure 1_Blots/Figure 1B Western blot_SPO16-input-co-IP.tif]

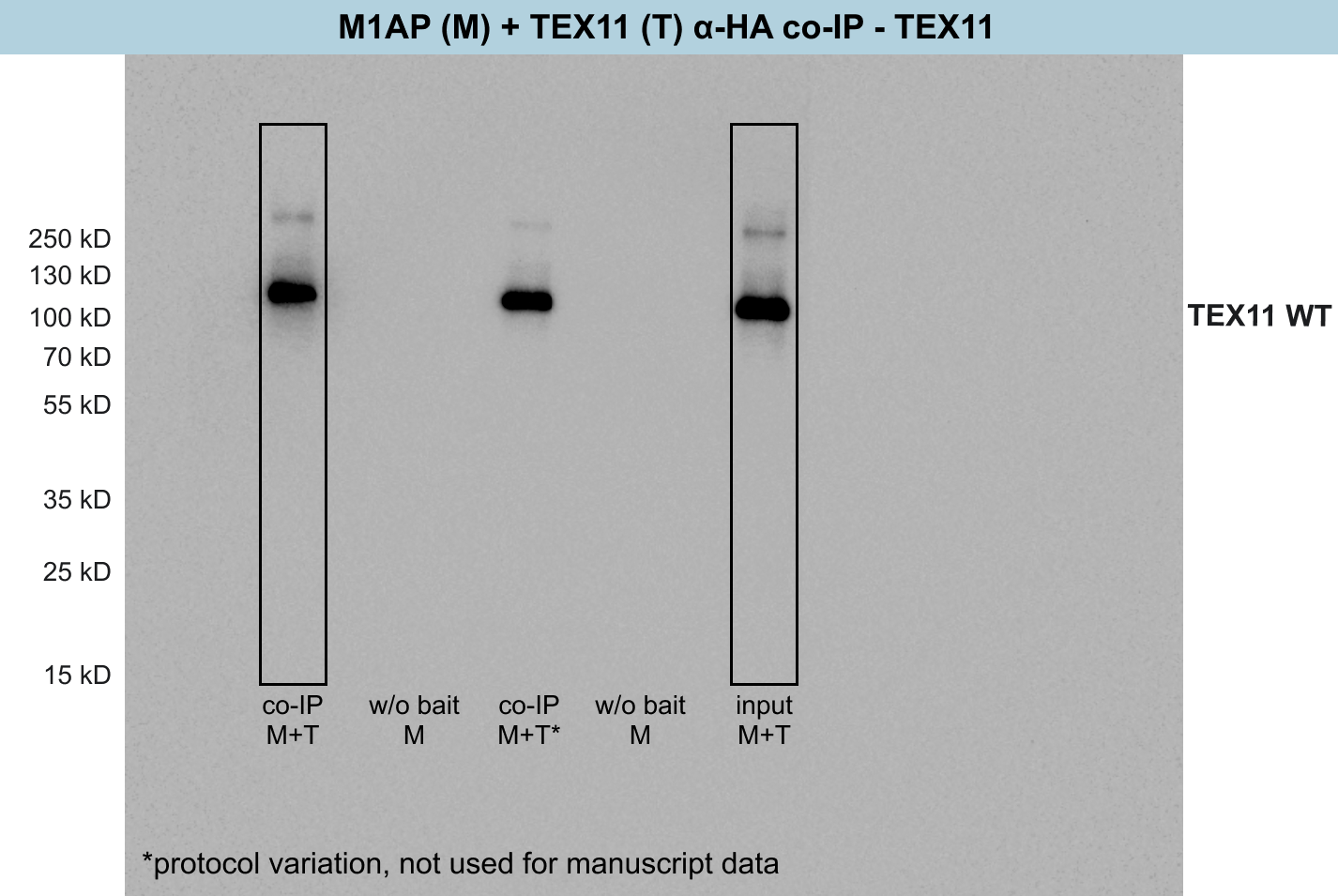

Supplement: Supplementary file 4 — Source data Fig. 1 [file 44321_2025_244_MOESM4_ESM.zip › Source Data_Figure 1_Blots/Figure 1B Western blot_TEX11-input-co-IP.tif]

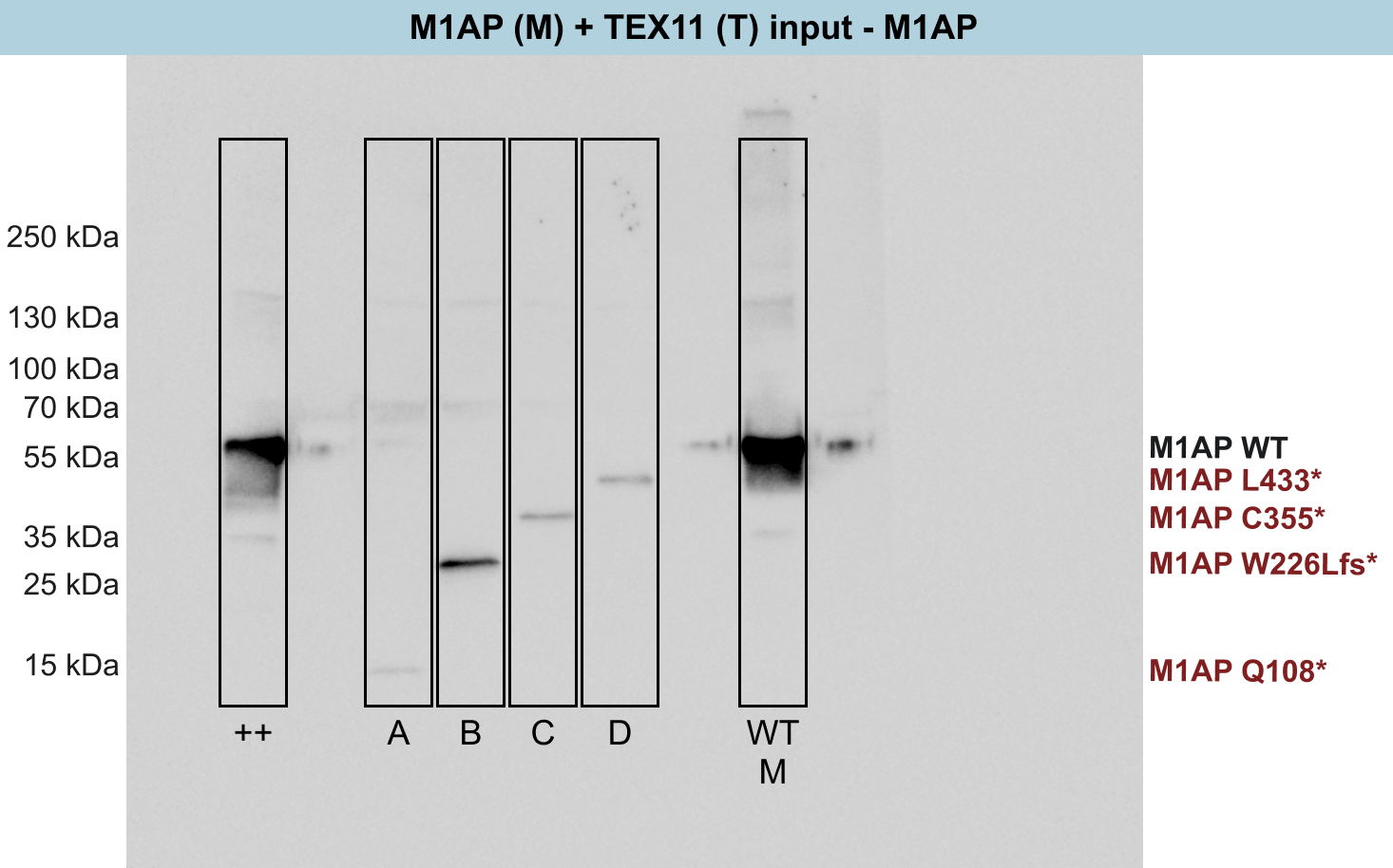

Supplement: Supplementary file 12 — Figure EV1 Source Data [file 44321_2025_244_MOESM12_ESM.zip › Source Data_Figure EV1/Figure EV1A_M1AP_input.tif]

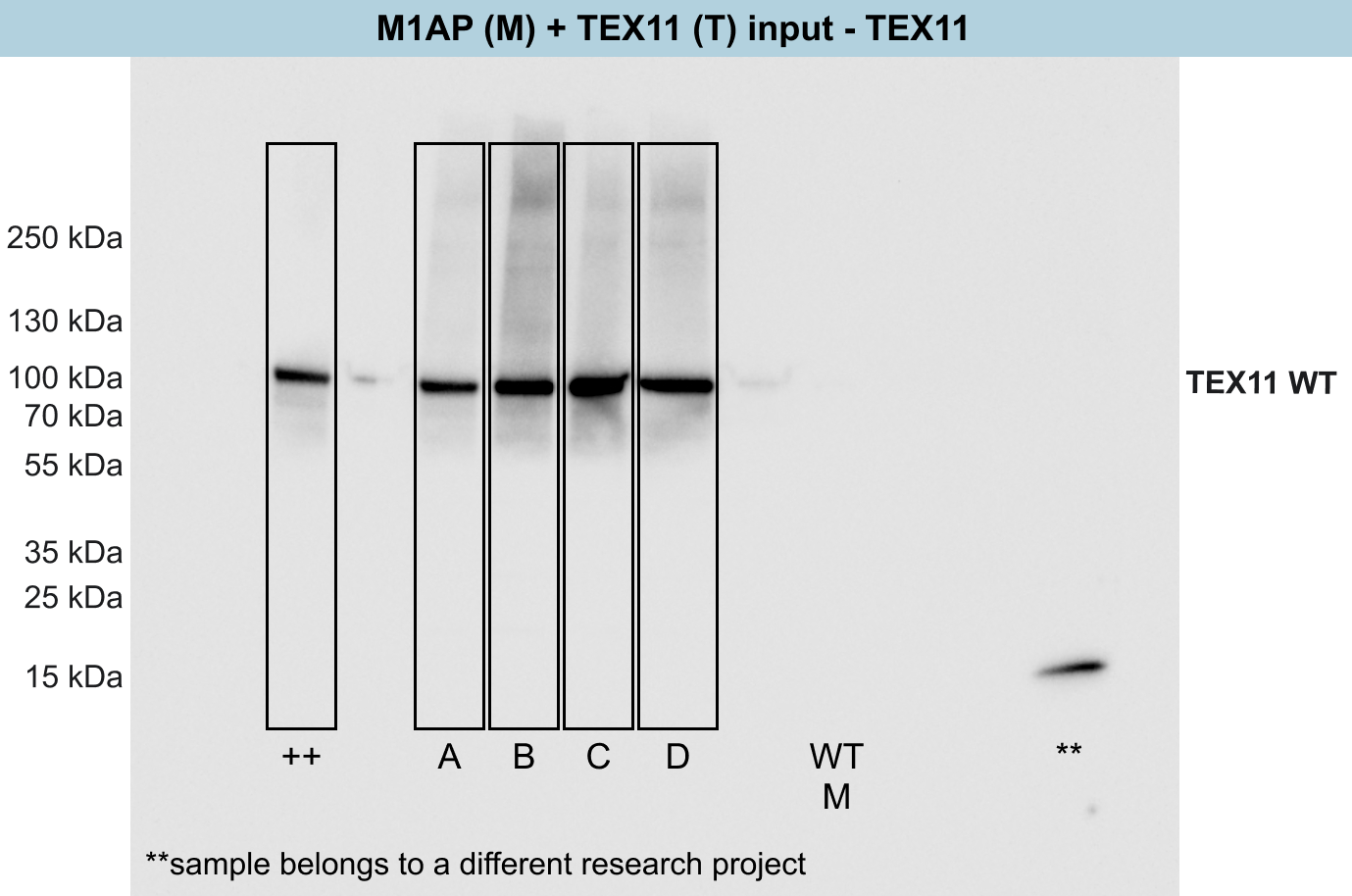

Supplement: Supplementary file 12 — Figure EV1 Source Data [file 44321_2025_244_MOESM12_ESM.zip › Source Data_Figure EV1/Figure EV1A_TEX11_input.tif]

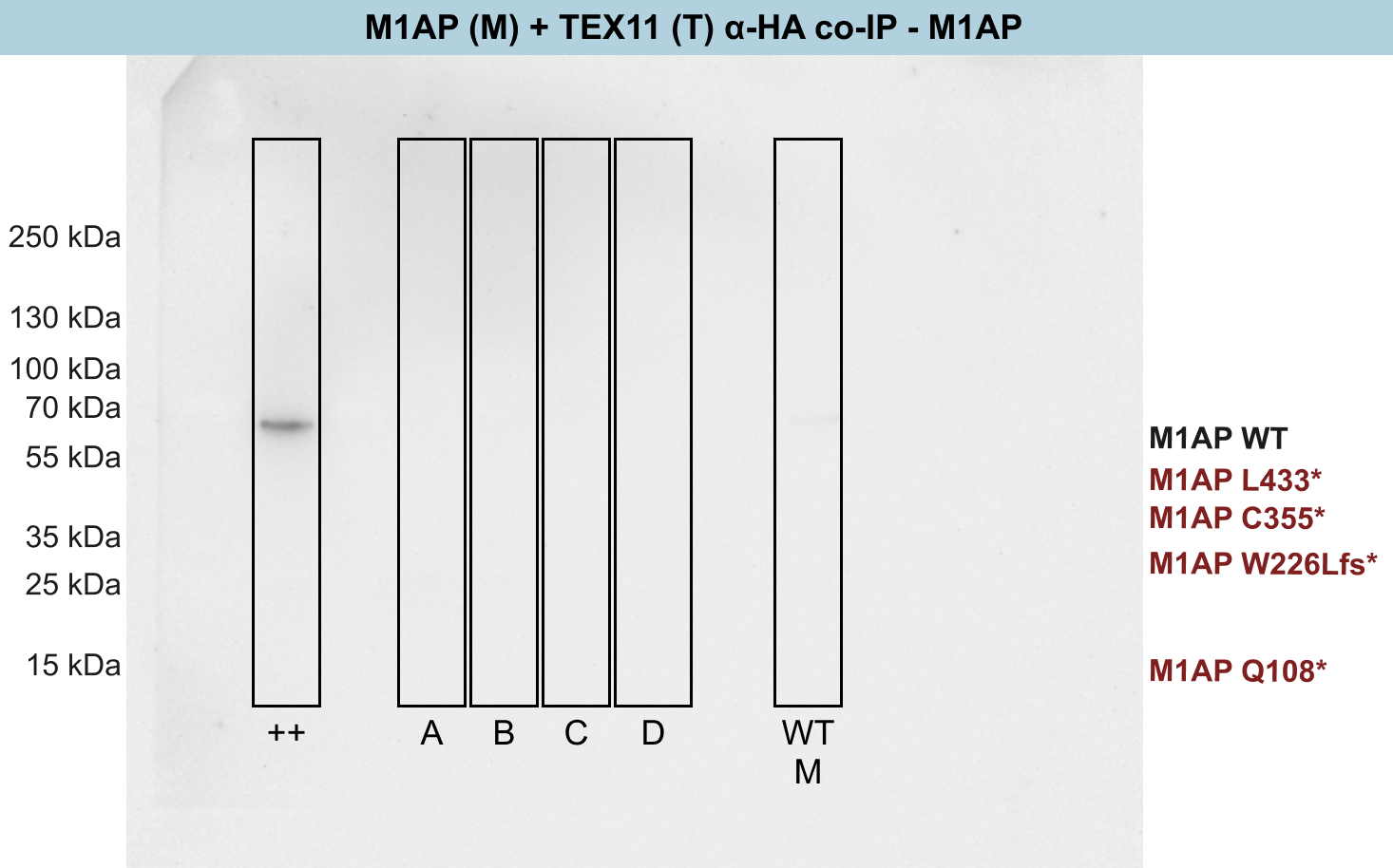

Supplement: Supplementary file 12 — Figure EV1 Source Data [file 44321_2025_244_MOESM12_ESM.zip › Source Data_Figure EV1/Figure EV1B M1AP co-IP.tif]

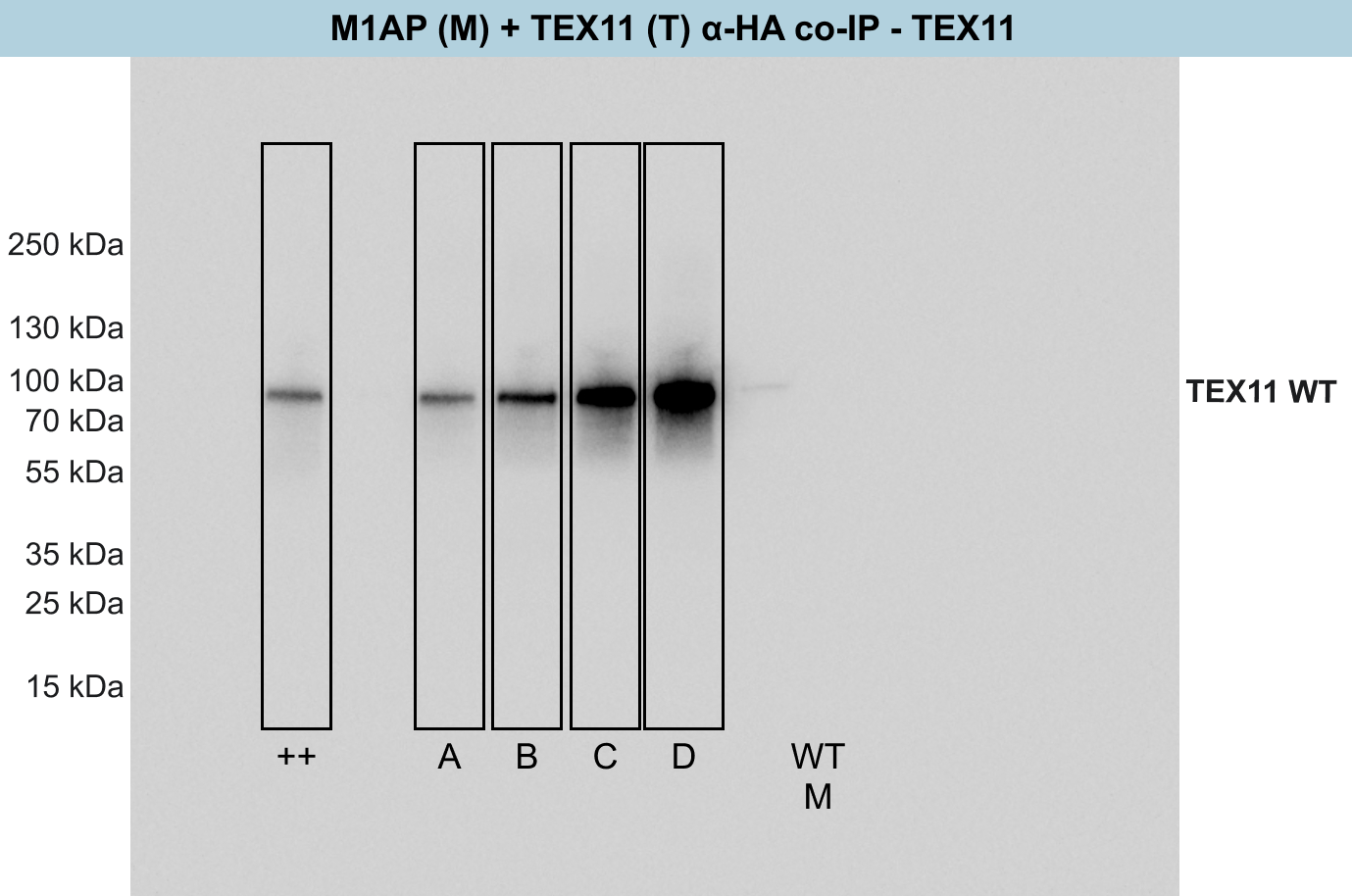

Supplement: Supplementary file 12 — Figure EV1 Source Data [file 44321_2025_244_MOESM12_ESM.zip › Source Data_Figure EV1/Figure EV1B_TEX11_co-IP.tif]
