# Supplementary material for: Genotype-specific differences in infertile men due to loss-of-function variants in M1AP or ZZS genes
Source: EMBO Mol Med. 2025 May 15;17(6):1417–51. doi: 10.1038/s44321-025-00244-0 (PMC12162868; doi:10.1038/s44321-025-00244-0)
Supplement: Supplementary file 13 — Expanded View Figures [file 44321_2025_244_MOESM13_ESM.pdf]

Expanded View Figures

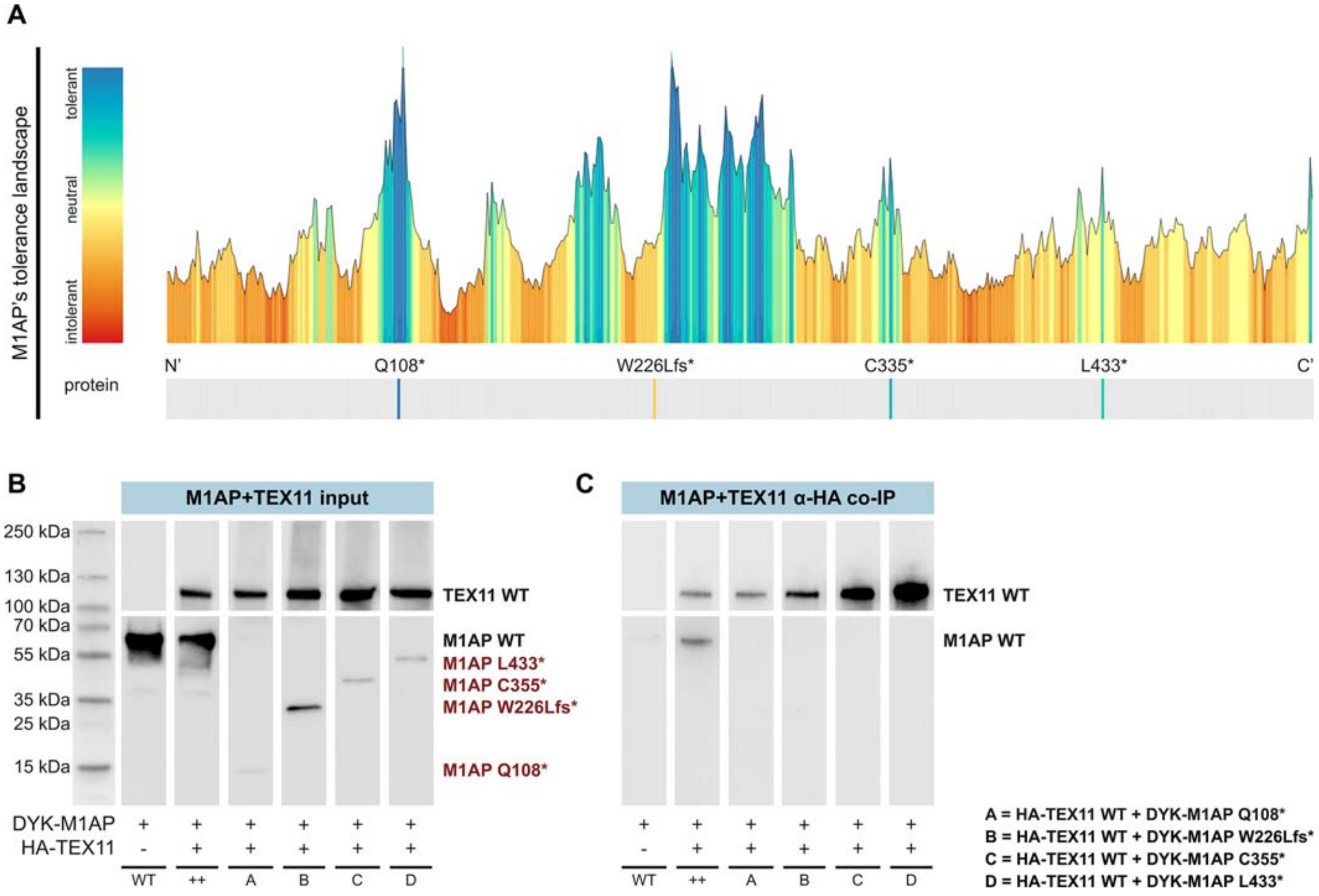

Figure EV1. Full-length M1AP is mandatory for the protein-protein interaction with TEX11.

(**A**) The protein tolerance landscape of M1AP illustrates the respective regions selected for mutagenesis for cloning truncated versions (Q108*: c.322 C > T p.Gln108Ter, C335*: c.1005 T > A p.Cys335Ter, L433*: c.1297 C > T, c.1298 T > A p. Leu433Ter). Positions were selected in tolerant regions (blue, green) of M1AP to prevent destruction of the protein's function. The construct c.676dup p.W226L*4 has been described in (Wyrwoll et al, 2020). (**B**) Western blot analysis of the input lysates of the co-transfection of full-length TEX11 (WT, detected by C-terminal HA tag) with full-length (WT) and truncated M1AP constructs (detected by N-terminal DYK-tag) confirm the expression in HEK293T cells. (**C**) Co-immunoprecipitation (IP) proved the interaction of human WT M1AP with WT TEX11. In contrast, no truncated M1AP was detected upon co-transfection with TEX11, pointing towards an absence of protein-protein interaction and thereby specifying the M1AP-TEX11 WT interaction. Experiments were replicated in three biological replicates. Source data are available online for this figure.

 © The Author(s)

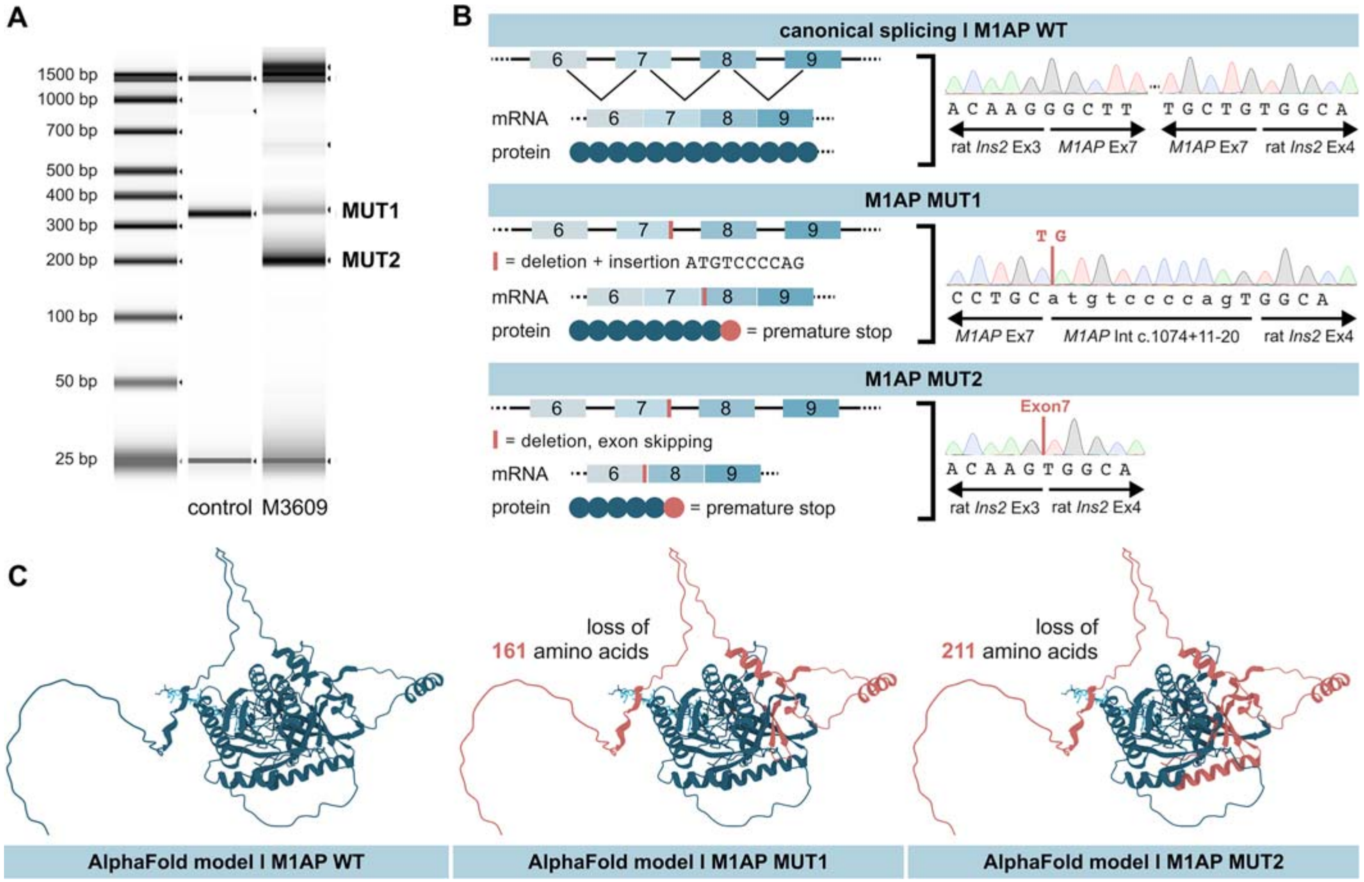

Figure EV2. ***M1AP* splice site variant identified in M3609.**

(**A**) Amplified minigene cDNA encompassing—c.1073_1074+10del or respective wild-type (control/WT) sequence. (**B**) Schematic illustration of variant effect on genomic, transcriptomic, and protein level combined with sequencing results for each minigene product reveals aberrant splicing. In the WT minigene construct, M1AP exon 7 (Ex7) is encompassed by two known exons of rat Insulin 2, exon 3 and exon 4 (rat Ins2 Ex3/Ex4). In M3609, the variant led to two splicing products: one (MUT1) showed the recognition of a cryptic splice site leading to a frameshift and premature stop codon in M1AP exon 8. The second (MUT2) resulted in skipping of exon 7 and a premature stop codon in exon 8. (**C**) Both splicing products lead to the loss of amino acids, presumably effecting M1AP's function and interaction.

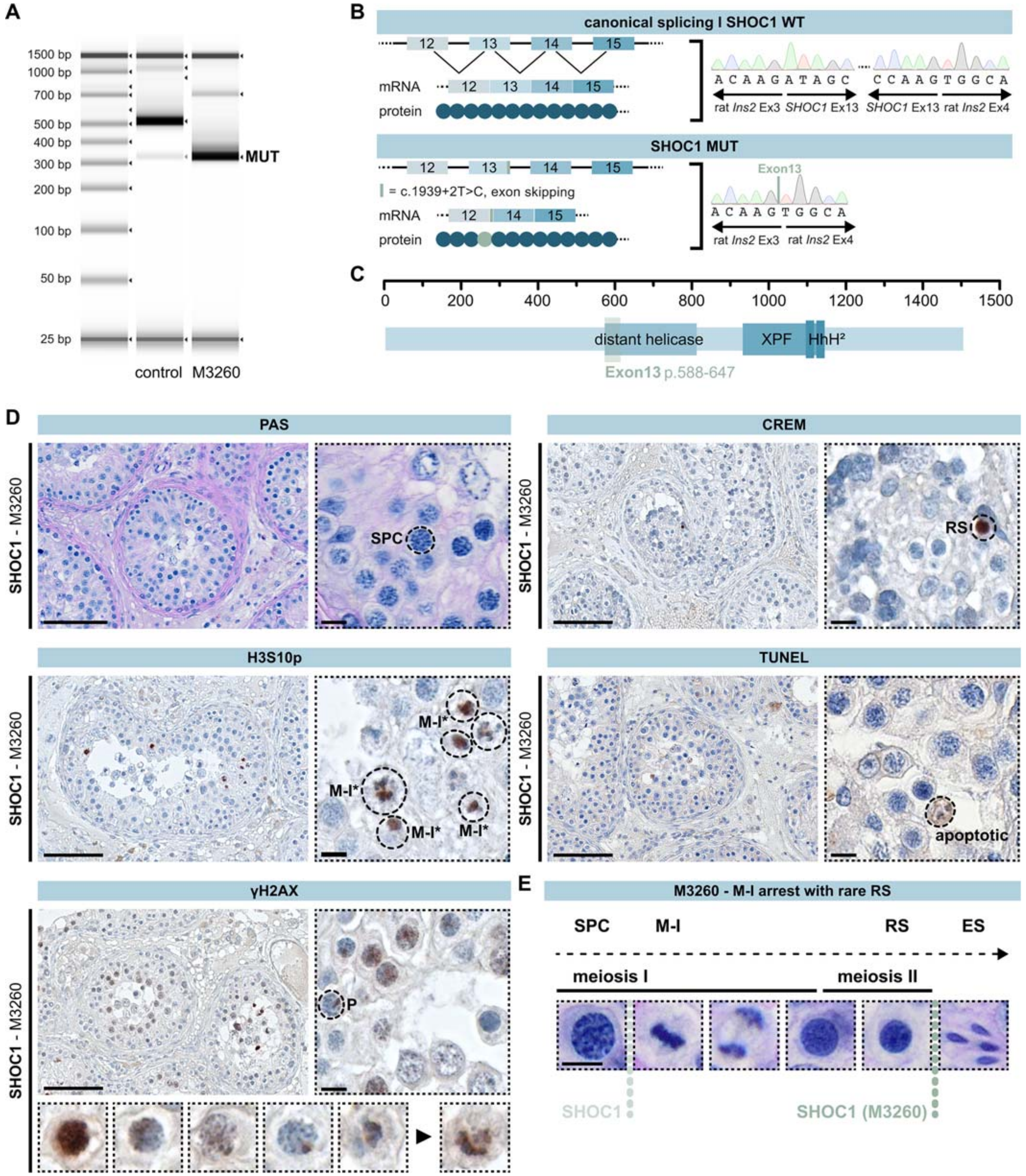
A
1500 bp
1000 bp
700 bp
500 bp
400 bp
300 bp
200 bp
100 bp
50 bp
25 bp
MUT
control M3260
B
canonical splicing I SHOC1 WT
12 13 14 15
mRNA 12 13 14 15
protein
ACAAGATAGC CCAAGTGGCA
rat Ins2 Ex3 SHOC1 Ex13 SHOC1 Ex13 rat Ins2 Ex4
SHOC1 MUT
12 13 14 15
= c.1939+2T>C, exon skipping
mRNA 12 14 15
protein
Exon13
ACAAGTGGCA
rat Ins2 Ex3 rat Ins2 Ex4
C
0 200 400 600 800 1000 1200 1400 1500
distant helicase XPF HhH²
Exon13 p.588-647
D
PAS
SHOC1 - M3260
SPC
CREM
RS
H3S10p
M-I*
TUNEL
apoptotic
γH2AX
P
E
M3260 - M-I arrest with rare RS
SPC M-I RS ES
meiosis I meiosis II
SHOC1 SHOC1 (M3260)

Figure EV3. *SHOC1* splice site variant identified in M3260 with predominant meiotic arrest and rare round spermatids.

(**A**) *SHOC1* (NM_173521.5) has 26 exons and its corresponding protein comprises 1444 amino acids. Amplified minigene cDNA encompassing—c.1939+2 T > C (M3260) or respective wild-type (control/WT) sequence. (**B**) Schematic illustration of variant effect on genomic, transcriptomic, and protein level combined with sequencing results for each minigene product reveals aberrant splicing. In the WT minigene construct, *SHOC1* exon 13 (Ex13) is encompassed by two known exons of rat *Insulin 2*, exon 3 and exon 4 (rat *Ins2* Ex3/Ex4). In M3260, the variant resulted in in-frame skipping of exon 13 and a predictive loss of 59 amino acids representing 4% of the total protein. (**C**) This affects the distant helicase hits region but not the highly conserved 'SHOC1 homology region' (amino acids 937–1105, NP_775792; Macaisne et al, 2008). This region contains an XPF endonuclease-like central and a helix-hairpin-helix (HhH^2) domain and is important for the XPF-ERCC1-like complex formation between SHOC1 and SPO16 (De Muyt et al, 2018; Zhang et al, 2019). Yeast studies highlighted that the N-terminal part of Zip2 is linked to the chromosome axis and the other ZMM components through Zip4 interaction, while the XPF domain interacts exclusively with Spo16 (De Muyt et al, 2018). Given that M3260 expresses all exons of *SHOC1* except for exon 13, the interaction with SPO16 and in parts with the ZMM proteins, such as TEX11, remains intact. However, a changed protein conformation due to the loss of exon 13 could influence some of these interactions and explain the observed testicular phenotype (**D**) of predominant meiotic arrest with rare round spermatids that were positive for CREM-staining. H3S10 staining showed only aberrant metaphase I-like spermatocytes (M-I*). TUNEL staining showed an increased number of apoptotic spermatocytes similar to patients with complete LoF variants in *M1AP, SHOC1, TEX11*, or *SPO16*. In γH2AX staining, single tubules contained pachytene-like cells (P) with a clearly distinguishable XY body were observed, which is in line with the presence of round spermatids. In addition, also aberrant pachytene-like cells were present. (**E**) The specific type of arrest of M3260 is described as a metaphase I arrest (MM-I) with rare round spermatids (RS) (panel taken from Fig. 2C). The scale bar represents 100 µm and 10 µm, respectively.

© The Author(s)

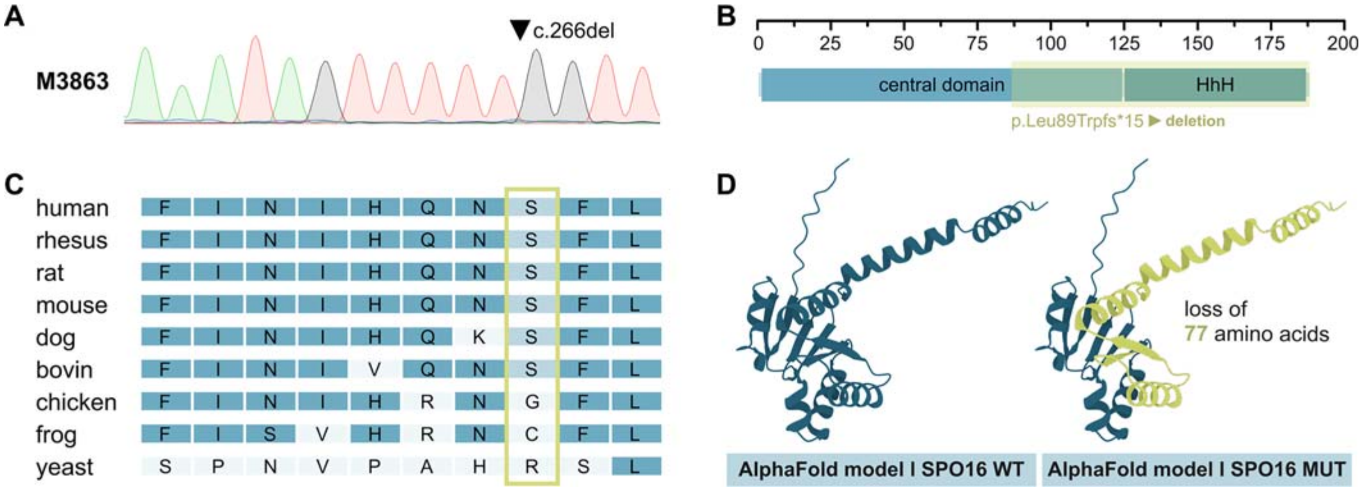

Figure EV4. ***SPO16*** **loss-of-function variant identified in M3863.**

(**A**) Sanger sequencing of M3863 revealed the frameshift variant c.266del leading to premature stop codon (p.Leu89Trpfs*15). (**B**) Such a truncated protein would lack the highly conserved helix-hairpin-helix (HhH^2) domain, which is important for the XPF-ERCC1-like complex formation between SHOC1 and SPO16 (De Muyt et al, 2018; Zhang et al, 2019). (**C**) Conversation analysis of the SPO16 variant. (**D**) The premature stop codon would truncate 42.5% of the complete protein.

 © The Author(s)

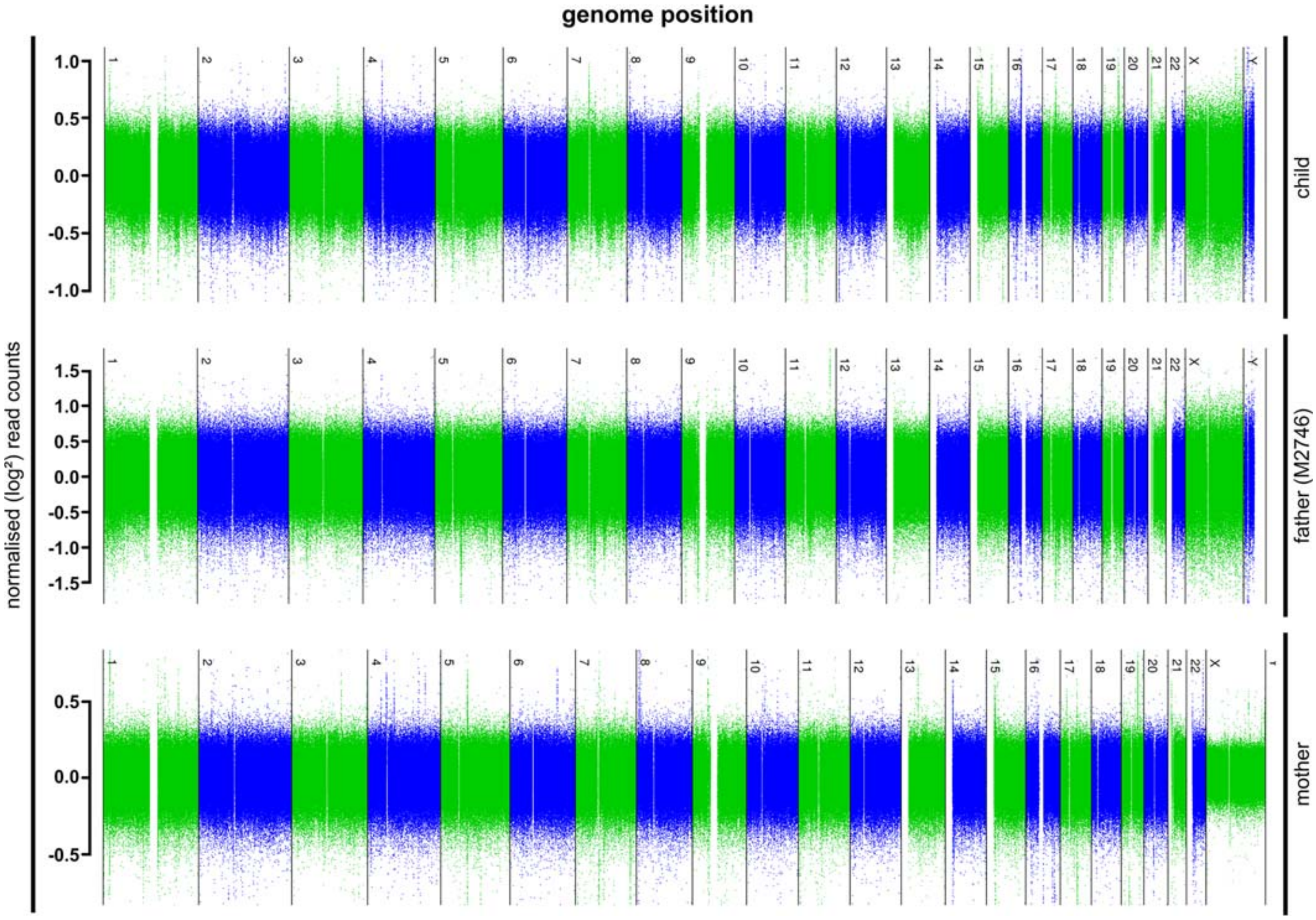

Figure EV5. Euploidy analysis of M2746, his child, and the child's mother.

Genome sequencing data was queried and read counts were normalised by dividing the median read count of each chromosome by the median read count of all autosomes. Normalised (log^2) read counts of autosomes (0.97 to 1.06) and of gonosomes (0.49 to 0.51) gave no evidence for chromosome aneuploidies.
